# Sources, Occurrence and Characteristics of Fluorescent Biological Aerosol Particles Measured Over the Pristine Southern Ocean

**DOI:** 10.1029/2021JD034811

**Published:** 2021-06-09

**Authors:** Alireza Moallemi, Sebastian Landwehr, Charlotte Robinson, Rafel Simó, Marina Zamanillo, Gang Chen, Andrea Baccarini, Martin Schnaiter, Silvia Henning, Robin L. Modini, Martin Gysel‐Beer, Julia Schmale

**Affiliations:** ^1^ Laboratory of Atmospheric Chemistry Paul Scherrer Institute Villigen Switzerland; ^2^ Extreme Environments Research Laboratory École Polytechnique Fédérale de Lausanne, School of Architecture, Civil and Environmental Engineering Lausanne Switzerland; ^3^ Remote Sensing and Satellite Research Group Curtin University Bentley WA Australia; ^4^ Institut de Ciències del Mar (CSIC) Barcelona Spain; ^5^ Institut für Meteorologie und Klimaforschung Karlsruher Institut für Technologie Karlsruhe Germany; ^6^ schnaiTEC GmbH Bruchsal Germany; ^7^ Leibniz Institute for Tropospheric Research, Experimental Aerosol and Cloud Microphysics Leipzig Germany

**Keywords:** Atmospheric aerosols, bioaerosols, fluorescent aerosols, marine aerosols, sea spray aerosols, Southern Ocean

## Abstract

In this study, we investigate the occurrence of primary biological aerosol particles (PBAP) over all sectors of the Southern Ocean (SO) based on a 90‐day data set collected during the Antarctic Circumnavigation Expedition (ACE) in austral summer 2016–2017. Super‐micrometer PBAP (1–16 µm diameter) were measured by a wide band integrated bioaerosol sensor (WIBS‐4). Low (3σ) and high (9σ) fluorescence thresholds are used to obtain statistics on fluorescent and hyper‐fluorescent PBAP, respectively. Our focus is on data obtained over the pristine ocean, that is, more than 200 km away from land. The results indicate that (hyper‐)fluorescent PBAP are correlated to atmospheric variables associated with sea spray aerosol (SSA) particles (wind speed, total super‐micrometer aerosol number concentration, chloride and sodium concentrations). This suggests that a main source of PBAP over the SO is SSA. The median percentage contribution of fluorescent and hyper‐fluorescent PBAP to super‐micrometer SSA was 1.6% and 0.13%, respectively. We demonstrate that the fraction of (hyper‐)fluorescent PBAP to total super‐micrometer particles positively correlates with concentrations of bacteria and several taxa of pythoplankton measured in seawater, indicating that marine biota concentrations modulate the PBAP source flux. We investigate the fluorescent properties of (hyper‐)fluorescent PBAP for several events that occurred near land masses. We find that the fluorescence signal characteristics of particles near land is much more variable than over the pristine ocean. We conclude that the source and concentration of fluorescent PBAP over the open ocean is similar across all sampled sectors of the SO.

## Key Points

- Fluorescent primary bioaerosol particles (PBAP) were measured over all sectors of the Southern Ocean
- Moderate to good correlations were observed between PBAP and sea spray aerosol (SSA) proxies
- PBAP fractions in SSA were positively correlated to concentrations of certain marine biota

## 1 Introduction

Primary biological aerosol particles (PBAP) are ubiquitous atmospheric particles emitted from the biosphere, which encompass intact microorganisms (e.g., bacteria, viruses, pollen, fungal spores etc.), or fragments of such microorganisms (Després et al., 2012; Fröhlich‐Nowoisky et al., 2016). PBAP have major impacts on public health, as certain types of PBAP are known to act as allergens or spread disease (Després et al., 2012; Fröhlich‐Nowoisky et al., 2016; Taylor et al., 2004). Furthermore, long‐range transport of PBAP, such as bacteria, could influence the ecosystem and biome diversity of the environments to which they are transported (Burrows et al., 2009; Hervàs et al., 2009; Kellogg & Griffin, 2006). Moreover, PBAP have the potential to affect cloud formation, for example by acting as giant cloud condensation nuclei (Pope, 2010) at low supersaturations. A number of studies have demonstrated that PBAP are effective ice nucleating particles (INP) (Després et al., 2012; Tobo et al., 2013), thereby facilitating glaciation of super‐cooled liquid clouds via heterogeneous ice nucleation (Kanji et al., 2017). Such aerosol‐cloud interactions can modify cloud optical properties and precipitation patterns with important atmospheric impacts on regional and global scales (Kanji et al., 2017).

PBAP originate from both the terrestrial and marine biosphere (Després et al., 2012). In the oceanic environment, primary aerosol particles, known as sea spray aerosol (SSA) particles, are produced through a combination of processes, which includes breaking of waves, generation of bubbles in the oceanic water, rising of bubbles to the ocean surface and the subsequent bubble bursting and aerosol ejection (de Leeuw et al., 2011; Lewis & Schwartz, 2004). Additionally, larger sea spray droplets known as spume droplets can be torn directly from wave crests during strong wind conditions (Monahan et al., 1986). In addition to inorganic sea salt, SSA consists of complex arrays of organic compounds (Brooks & Thornton, 2018; Hawkins & Russell, 2010; O'Dowd & de Leeuw, 2007; Prather et al., 2013). SSA organic compounds have their origin in seawater dissolved organic matter (DOM) (Hawkins & Russell, 2010), particulate organic matter (POM) such as polysaccharides and proteinaceous gel‐like particles (Aller et al., 2017), and microorganisms such as bacteria, viruses and phytoplankton (Quinn et al., 2015). Studies on the chemical composition of laboratory‐generated SSA indicate that seawater bioactivity influences the fraction of organic matter in SSA by altering the abundance of microorganisms in water (Ault et al., 2013; Lee et al., 2020; Wang et al., 2015). Laboratory mesocosm experiments have also shown that marine microbes themselves can be transferred into SSA (McCluskey et al., 2017). In addition to laboratory‐based studies, analysis of aerosol samples collected in different global oceanic regions have demonstrated that marine microorganisms and associated organic components are incorporated into SSA (e.g., Ceburnis et al., 2016; Mayol et al., 2017; Orellana et al., 2011; Russell et al., 2010). More recently, sequencing analysis of aerosol samples from the Southern Ocean (SO) also demonstrated that bacteria were present in the SSA (Uetake et al., 2020). These studies indicate that PBAP contribute to SSA‐associated primary organic matter.

Previous studies indicate that some SSA particles possess ice nucleating properties (Bigg, 1973; Schnell & Vali, 1976), and it was suggested that this could be related to marine biological activity. More recent studies have demonstrated that SSA containing both dissolved and/or particulate organic matter are capable of nucleating ice crystals efficiently at temperatures in the range −20°C to −35°C (DeMott et al., 2016; McCluskey et al., 2018; Wang et al., 2015; Wilbourn et al., 2020; Wilson et al., 2015). Such SSA particles tend to nucleate ice at lower temperatures than their terrestrial counter‐parts, that is, they are less effective INP (DeMott et al., 2010), which necessitates the segregation of terrestrial and marine INP parametrizations in global atmospheric models (Vergara‐Temprado et al., 2017). Overlooking such a distinction in INP parametrizations can increase the uncertainty in global atmospheric models.

The Southern Ocean (SO) is a pristine environment (e.g., Hamilton et al., 2014; Schmale et al., 2019) as well as the roughest ocean on Earth in terms of surface winds and waves (Young, 1999). This makes the SO an extremely promising location to study SSAs and their associated PBAP. However, our knowledge regarding the regional distribution and composition of SO SSA and PBAP is still very limited (Middlebrook et al., 1998; Murphy et al., 1998; Uetake et al., 2020). In addition, studies have indicated considerable uncertainties in calculated radiative forcing over the SO (Flato et al., 2013). These uncertainties are partly attributed to misrepresentation of SO aerosol and associated processes, for example, excessive heterogeneous ice crystal formation and subsequent precipitation in global atmospheric models (Vergara‐Temprado et al., 2017, 2018). Considering the unique properties of marine PBAP and their potential effects on cloud microphysics, identification, quantification, and source apportionment of these particles is an important step toward improving the representation of SO aerosols in global climate models.

Identification and quantification of atmospheric PBAP of oceanic origin is prone to several challenges. Conventional methods rely on atmospheric sample extraction and offline analysis (Després et al., 2012; Fröhlich‐Nowoisky et al., 2016). Although the analysis of offline samples can provide detailed morphological, chemical and biological information on PBAP, it remains time consuming. This limits the obtainable sample sizes through offline analysis, making it difficult to gain quantitative insights. In addition, the relatively poor time resolution of offline samples complicates source identification.

More recently, online PBAP detection methods based on aerosol auto‐fluorescent properties have become available (e.g., Fennelly et al., 2018). Online PBAP detection methods typically rely on ultra‐violet light induced fluorescence (UV‐LIF). These methods employ UV excitation of single particles, followed by spectrally resolved or waveband integrated detection of the resulting fluorescent light. The wavelength detection ranges are chosen to match regions of fluorescence for biological compounds that are found ubiquitously in PBAP, such as tryptophan and Nicotinamide Adenine Dinucleotide (NADH) (Fennelly et al., 2018; Kaye et al., 2005). To date, online PBAP measurements have been employed in both laboratory studies (Hernandez et al., 2016; Savage et al., 2017) and field measurements (Crawford et al., 2016, 2017; Healy et al., 2014; Perring et al., 2015; Pöhlker et al., 2012; Toprak & Schnaiter, 2013; Ziemba et al., 2016). In the context of field measurements, the key advantage of online UV‐LIF techniques is that they facilitate size‐resolved quantitative measurements of PBAP concentrations at high time resolution. This makes it possible to compare them to other highly variable environmental parameters, thereby facilitating identification of PBAP sources. To the best of our knowledge, only two studies have used online UV‐LIF methods to investigate PBAP in the Antarctic and SO regions (Crawford et al., 2017; McFarquhar et al., 2020). Crawford et al. (2017) identified fluorescent particles measured in the Halley VI station along the Antarctic coast as dust and/or pollen particles transported from the Antarctic and South American continents, or as PBAP transported from biologically active coastal marginal ice zones. However, it is not clear if these results are representative of other SO regions, particularly remote oceanic regions far from continental influence. McFarquhar et al. (2020) report median PBAP concentrations measured during the Measurements of Aerosols, Radiation, and Clouds over the Southern Ocean research cruise (MARCUS, October 2017 – April 2018) with no additional analyses of possible sources and sinks. Therefore, further online, fluorescence‐based measurements are required to gain better insights into PBAP over the SO.

In the current study, we strive to explore the occurrence and origin of marine PBAP in the pristine SO region with an extensive database of new measurements. Co‐located marine and atmospheric measurements were performed during the research cruise Antarctic Circumnavigation Expedition (ACE) between December 2016 and March 2017 (Schmale et al., 2019), including online auto‐fluorescence measurements of PBAP made with a wideband integrated bioaerosol sensor (WIBS‐4). This unique data set represents one of the largest sets of aerosol measurements ever collected over all sectors of the SO. Section 2 describes the details of the data set, instrumentation and data analysis assumptions. We investigate the link between PBAP and SSA in Sections 3.1 and 3.2. Additionally, a comprehensive set of measurements of seawater chemical composition and biological activity were conducted during ACE. In Section 3.3, we compare the variability of the seawater measurements to that of the fluorescent aerosols in order to explore the ocean‐originating source of the PBAP. Finally, we present the spatial concentration distribution, and microphysical and fluorescent properties of PBAP in Sections 3.4, 3.5, 3.6, 3.7, 3.8. Overall, this study provides comprehensive insights into the distribution of SSA‐related PBAP over the SO, and sheds light on the marine biological components responsible for the observed PBAP.

## 2 Materials and Methods

### 2.1 Campaign Description

**Figure 1:**
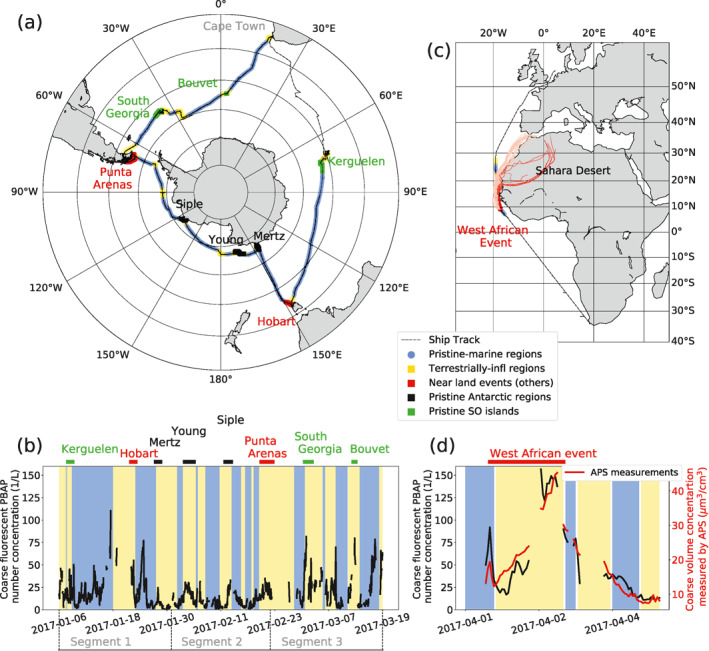
(a) Map of the investigated segments (Segments 1, 2, and 3) of the Antarctic Circumnavigation Expedition (ACE) cruise. The map shows regions defined as pristine‐marine in blue (further than 200 km from land masses) and terrestrially influenced in yellow (closer than 200 km to land masses). Several terrestrially influenced regions where relatively strong fluorescent particle events occurred are also shown in the figure, and these are further classified into pristine Southern Ocean (SO) islands (green), pristine Antarctic (black), and near populated continental regions events (red). (b) Time series of the fluorescent primary biological aerosol particles (PBAP) number concentration measured during the ACE campaign (3σ threshold). The time periods of pristine‐marine (blue shade), terrestrially influenced (yellow shade) and other selected events are highlighted in the time series. (c) Map of the return path along West Africa where a dust plume was intercepted by the ship (red region). Five‐day air mass back trajectories calculated with the Lagrangian analysis tool LAGRANTO (Sprenger & Wernli, 2015; Thurnherr et al., 2020). The back trajectories corresponding to air masses passing over the Saharan Desert are shown as red lines while air masses from other regions are shown as pink lines. The back trajectories during the event indicate that air traveled south along West Africa and the Saharan Desert before reaching the ship. (d) Time series of fluorescent PBAP number concentrations and total coarse aerosol volume concentrations (measured by the APS) for the West African section shown in (c). The blue shades correspond to pristine‐marine time periods, the yellow shades are the terrestrially influenced and the red line is the presumed dust event.

We acquired the results presented in this study during ACE conducted from December 2016 to March 2017 (Schmale et al., 2019). A detailed overview of the ACE campaign can be found in the cruise report (Walton & Thomas, 2018). In this campaign, we performed co‐located marine and atmospheric measurements aboard the research vessel *Akademik Tryoshnikov*. ACE covered an extensive range of geographical locations (Figure 1) starting from Cape Town, South Africa, and circumnavigating the SO before returning back to Cape Town. To simplify the geographical extent for analyses in this work, we divided the route into three segments. Segment 1 represents samples collected from January 6, 2017 to January 31, 2017, which covers the route from Kerguelen Islands to the Mertz Glacier in Antarctica. Segment 2 represents samples collected from January 31, 2017 to February 22, 2017, which covers the track from the Mertz Glacier to Punta Arenas in Chile. Segment 3 represents samples collected from February 22, 2017 to March 19, 2017, which covers the area between Punta Arenas and Cape Town. Although the campaign started in Cape Town, we used only those data acquired after the Kerguelen Islands because the internal pump of the instrument did not function properly at the beginning of the cruise. This pump was replaced by an external pump during the station at Kerguelen Islands. It should be noted that in this study the campaign route is divided differently than in previously published ACE studies (Schmale et al., 2019), in which divided segments are referred to as *legs*. Additionally, Figure 1c shows the ship return path from Cape Town to Bremerhaven, Germany, along the west coast of Africa, where a Saharan dust plume was likely intercepted by the ship based on high aerosol loads and modeled air mass back trajectories. We compared the results from this period against the SO measurements in Section 3.3.

### 2.2 Fluorescent Aerosol Measurements

We used a wideband integrated bioaerosol sensor (WIBS‐4, University of Hertfordshire, Hatfield, UK) to measure fluorescent aerosol particles on a single‐particle basis. Moreover, the WIBS measures aerosol optical diameter in the size range from 0.5 to 15 μm, and an asymmetry factor (AF) which is a measure of aerosol morphology. Toprak and Schnaiter (2013) demonstrated that an AF value of 8 represents spherical particles, while higher AF values are associated with non‐spherical particles. The aerosol fluorescent measurements are conducted by exciting particles with two Xenon flash lamps at wavelengths of 280 and 370 nm and then detecting the fluorescent light intensity in the wavebands from 310‐400 nm and 420–650 nm. This results in three different excitation wavelength (ExWL) and emission waveband (EmWB) configurations: channel A (ExWL 280 nm and EmWB 310–400 nm), channel B (ExWL 280 nm and EmWB 420–650 nm) and channel C (ExWL 370 nm and EmWB 420–650 nm).

The fluorescent detection threshold in each channel is determined based on the background signal measured during a “forced triggering” (FT) procedure. In this study, two detection thresholds were considered: 3σ (FT mean plus 3 times the FT standard deviation) and 9σ (FT mean added plus 9 times the FT standard deviation). These thresholds have been previously applied by Savage et al. (2017). We distinguish the results obtained with these two different threshold settings by referring to them as the fluorescent particle (3σ) and hyper‐fluorescent particle (9σ) results. It is important to note that the hyper‐fluorescent particles are the subset of fluorescence particles displaying the strongest fluorescent signals. Moreover, we use the classification scheme introduced by Perring et al. (2015). In this method, the fluorescent particles are divided into 7 different classes (A, B, C, AB, AC, BC, and ABC) based on the logical combination of emitted signals in the three fluorescent channels. More details about WIBS and the ABC classification is provided in the supplementary information (Section S1.1).

During ACE, ambient air was drawn from a standard Global Atmospheric Watch air inlet (Weingartner et al., 1999) mounted onto a laboratory container where the WIBS was located along with other aerosol instruments. The height of the inlet was ∼15 m above sea level. The sampling line was heated to maintain the relative humidity of the sample air below 40% (see Section S1.2 for further details). To minimize the interference of fluorescent aerosols of non‐biological origin (e.g., fluorescent particulate matter in the ship exhaust) we used an empirical masking technique to remove samples that were suspected to be contaminated by ship exhaust (see Section S1.3 for further details). Approximately 33% of all measurements made during cruise segments 1–3 were removed after applying this mask. The removed data points were distributed fairly evenly over the cruise track and they tended to be located close to land masses.

### 2.3 Auxiliary Atmospheric Measurements Used as Proxies for SSA Concentrations

We use a range of auxiliary atmospheric measurements in this study as proxy variables for the concentration of SSA in the air. It is necessary to use proxies for the concentration of SSA since it is difficult to measure this parameter directly, due to the fact that it is difficult to isolate SSA from other aerosol types found in the marine atmosphere like non‐sea‐salt sulfates (e.g., Modini et al., 2015).

Wind speed is often used as an indicator for SSA since SSA source strength and number concentration depend strongly on wind speed through wave breaking (Lewis & Schwartz, 2004). Although wind speed is a useful indicator of SSA production, one must always keep in mind potential differences between wind speeds at the point of SSA production and wind speeds at the point of measurement (in this case the research vessel), which complicates SSA‐concentration‐wind‐speed relationships. Here we report wind speeds as 10‐m neutral wind speeds, which were derived from the on board measurements (including a correction for air‐flow distortion) as described in Landwehr et al. (2020).

The dominant inorganic chemical component of SSA is NaCl (e.g., Bates et al., 2008). Therefore, the concentrations of sodium and chloride are useful markers for SSA (e.g., Modini et al., 2015; Quinn et al., 2017). Sodium ion concentrations were measured for sub‐10 µm aerosols using ion chromatography, which was performed offline on filter samples that had been collected over 24 h (Tatzelt et al., 2020). Inorganic chloride concentrations (Chen et al., 2019) were measured by a time‐of‐flight aerosol chemical speciation monitor (ToF‐ACSM, Aerodyne Research, Inc.; Fröhlich et al., 2013). The ACSM is only sensitive to the non‐refractory, submicrometer fraction of the total aerosol (i.e., the fraction that undergoes flash vapourization at 600°C). Therefore, the ACSM is only able to detect a very small fraction of the total chloride in SSA. This signal can be easily overwhelmed by anthropogenic sources of non‐refractory chloride (e.g., ammonium chloride), which prevents the use of ACSM chloride as a marker for SSA in environments with strong continental or anthropogenic influences. In the remote SO such influences are largely absent, and we assume that ACSM chloride represents SSA chloride qualitatively well.

For the same reason of geographical remoteness, we also assume that the number concentration of particles with diameters larger than 1 µm is a good proxy variable for SSA concentrations. That is, we assume that super‐micrometer particles with optical diameter larger than 1 µm, hereafter referred to as coarse mode, are composed predominantly of SSA particles. This is a reasonable assumption to make in remote marine locations since there are no major sources of coarse mode particles other than SSA production (on a number basis). The number size distributions of total aerosol particles (i.e., both fluorescent and non‐fluorescent particles) was obtained from the elastic scattering measurements performed with the WIBS, and these were integrated over diameters greater than 1 µm to calculate super‐micrometer number concentrations. Coarse aerosol number size distributions (Schmale et al., 2019) were also measured by an Aerodynamic Particle Sizer (APS, TSI Inc., Model 3321). Integrated super‐micrometer number concentrations from the WIBS and APS correlated well during segments 1–3, lending confidence to the measurements from both instruments (Figure S2). The integrated number concentrations also correlated well for the subset of measurements acquired after the ACE campaign (i.e., during the passage from Cape Town back to Europe), but the absolute ratio between these two parameters was higher compared to the value measured during segments 1–3. This suggests a drift in one or both of these instruments. Therefore, we consider the WIBS data measured during the return passage from Cape Town to Europe to be more uncertain than the WIBS data measured during segments 1–3.

### 2.4 Oceanic Measurements

We used additional measurements from other ACE projects, No. 1 and 8 (Walton & Thomas, 2018) to investigate links between airborne fluorescent PBAP and seawater composition (including dissolved compounds and microbial characteristics).

Seawater from approximately 5 meter depth was sampled from an underway seawater supply and preserved for later analysis or measured on‐board. In this study, we use the measurements of microbial composition (phytoplankton taxa relative pigment biomass contributions) (Antoine et al., 2019), biomass (particulate organic carbon (Thomalla et al., 2020), total chlorophyll‐a concentration, and absorption by colored dissolved organic matter), microbial cell abundance (e.g., bacterial cell number concentration), and concentrations of transparent exopolymeric particles (TEP) and coomasie stainable particles (CSP) (measured as in Zamanillo et al., 2019).

A complete description of all ocean measurements is available in Supplementary Section S3 and Tables S2–S4, while the ACE cruise report (Walton & Thomas, 2018) provides further information on the objectives and sampling methods.

### 2.5 Data Analysis Considerations and Segregation of the Measurements

The main objective of this study is to investigate ocean‐derived fluorescent PBAP (i.e., those primary biological particles that are thought to be emitted with SSA). To isolate such particles we segregated our measurements into two main categories: *pristine‐marine* and *terrestrially influenced* samples. This segregation was performed based on proximity to land. Measurements that were performed within 200 km distance from any coastline (continental land mass or island) were classified as terrestrially influenced, while all other measurements were identified as pristine‐marine. The 200 km threshold was chosen by examining the coefficients of correlation between fluorescent particle number concentrations and three of the proxy variables for SSA concentrations (wind speed, total number of coarse mode particles, chloride concentration) as a function of the proximity to land. This analysis is shown in Figure S3 for the fluorescent particle category and Figure S4 for the hyper‐fluorescent particle category (as defined in Section 2.2). For both categories, the coefficients of correlation reach a plateau at a distance greater than approximately 200 km. Therefore, we chose this distance as the threshold to segregate pristine‐marine and terrestrially influenced samples.

It should be noted that other methods for segregating land‐influenced and oceanic samples are also possible. For example, air mass back trajectories could be used to perhaps obtain a clearer separation of the terrestrially influenced measurements. We did not apply this method in this study because it carries a greater risk that some terrestrially influenced samples are classified as pristine‐marine samples due to uncertainties in the calculated air mass back trajectories. Since our goal was to focus specifically on ocean‐derived particles, we instead opted for the simple but conservative threshold value of 200 km from any land mass. The corollary of this approach is that our terrestrially influenced category likely also contains a sizable fraction of pristine‐marine measurements, which we deemed to be an acceptable consequence since mixed marine‐terrestrial aerosols are not the focus of our study. At the same time, this approach provides a good estimate of the radius of influence of terrestrial PBAP sources.

We segregated the aerosol fluorescence measurements by optical particle diameter as measured by the WIBS‐4. In particular, we categorized the measurements into *fine* (optical diameter < 1 µm) and *coarse* (optical diameter > 1 µm) aerosol categories. Our main focus is on the coarse particles since: (a) larger particles are less likely to be long‐range transported and can therefore be more confidently attributed to local, oceanic sources; (b) any contamination particles such as soot remaining after application of the ship exhaust post‐processing filters described in Section 2.2 are more likely to reside in the fine category than the coarse category; and (c) the WIBS counting efficiency deteriorates for particles with diameters less than 0.7 µm (Healy et al., 2012). The consequence of our decision to focus on coarse particles is that we possibly exclude certain types of PBAP, for example, bacteria with sizes below 1 µm (Fröhlich‐Nowoisky et al., 2016).

## 3 Results and Discussion

### 3.1 Time Series of Fluorescent PBAP Number Concentrations Over the Campaign

Figure 1 presents the time series of coarse fluorescent PBAP number concentrations measured over the entire ACE campaign. During pristine‐marine conditions, fluorescent PBAP number concentrations varied considerably and ranged between 0.17 and 120.1 L^−1^. The median number concentration was 11.4 L^−1^ with the interquartile range (IQR) ranging between 5.6 L^−1^ to 21 L^−1^. The median number concentration of coarse hyper‐fluorescent PBAP was 0.87 L^−1^ with IQR ranging between 0.37 L^−1^ to 1.95 L^−1^. The corresponding concentrations in the terrestrially influenced regions were higher than those in the pristine‐marine regions. The median number concentration of fluorescent particles in the terrestrially influenced regions was 17.3 L^−1^ and the IQR ranging between 6.5 L^−1^ to 27.8 L^−1^. For hyper‐fluorescent particles under terrestrial influence, the median number concentration was 1.52 L^−1^ with IQR ranging between 0.58 L^−1^ and 2.9 L^−1^.

It is important to note the diversity of the terrestrial areas that contributed to the land‐influenced measurements. As shown by the cruise map and time series displayed in Figure 1 the terrestrially influenced samples comprised measurements that were performed near the continent of Antarctica, near pristine and unpopulated islands in the SO, and near the Australian (Hobart) and South‐American (Punta Arenas) continents. When the ship passed through terrestrially influenced regions close to uninhabited islands and coastal regions, as well as more populated continental areas, high peaks in concentrations of fluorescent PBAP, reaching up to 90 L^−1^, were occasionally observed. We visually identified nine of these high‐concentration events, as indicated in Figure 1: three occurred in the vicinity of pristine SO islands (Kerguelen, South Georgia, and Bouvet), three near continental Antarctica (Mertz Glacier, Young, and Siple Islands), and three near populated continental regions (Hobart, Punta Arenas, and West Africa on the return route). The highest fluorescent particle concentrations were measured during the West African event, when hourly averaged concentrations reached up to 160 L^−1^. The back trajectories of air masses within the marine boundary layer, which are included in Figure 1c, indicate that some air masses passed over the Saharan desert. In addition, Figure 1d shows the integrated aerosol volume concentration of coarse particles obtained from APS measurements for the West African event, indicating an increase in integrated volume concentration of aerosol particles during this period. Therefore, we identify the fluorescent particles measured during the West African event as Saharan dust particles. Although our main focus in this work is on pristine‐marine PBAP, the various different near‐land measurements provide an insightful contrast for the remote ocean measurements. Difference between near land events and pristine‐marine samples are further investigated through the measured fluorescence classes in Section 3.5.

### 3.2 Demonstration of the Link Between Fluorescent PBAP and SSA Particles in the Pristine‐Marine Atmosphere

Based on previous studies of SSA composition it is hypothesized that SSA production is the dominant source of PBAP in the remote oceanic regions far from land where the contribution of long‐range transported aerosol particles is less likely (see Section 1). To investigate this hypothesis, we assessed the level of correlation between measured fluorescent particle number concentrations and four proxy variables for SSA concentrations (Section 2.3; wind speed, total coarse aerosol number concentrations, and aerosol chloride and sodium mass concentrations). We used the combined results from segments 1–3 of the research cruise for this correlation analysis.

**Figure 2:**
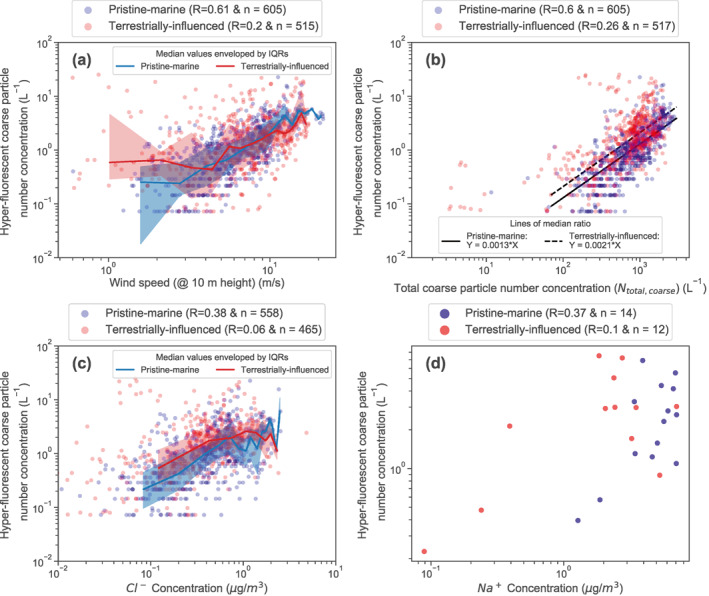
Scatter plots of pristine‐marine (blue) and terrestrially influenced (red) samples of hyper‐fluorescent particles versus four proxy variables for SSA concentrations: (a) wind speed, (b) total coarse particle concentrations, (c) chloride (Cl‐) concentrations as measured by the ACSM, and (d) sodium (Na+) concentrations measured offline from filter samples. Measurements from all segments are shown. The red and blue solid lines and shaded areas correspond to the medians and IQRs of the measurements, which were calculated by separating the data set into 10 equidistant logarithmic bins. Median lines and IQRs are not shown for the sodium ion measurements due to the small sample size. The number of tested samples for each condition (n) is included in the subplots.

Figure 2 presents scatter plots of hourly averaged hyper‐fluorescent PBAP number concentrations against the four variables (note Figure 2d presents 24h averaged measurements to match the filter sample collection periods). The results are split into pristine‐marine (blue points) and terrestrially influenced samples (red points) as described in Section 2.5. For the pristine‐marine samples, moderate correlation is observed between the hyper‐fluorescent number concentrations and all four proxies for SSA concentrations (Pearson's R values ranging from 0.37 to 0.61). These results suggest that the same underlying process drives the variability in all of these measured quantities, which supports the hypothesis that sea spray is an important source of fluorescent PBAP in the pristine‐marine atmosphere.

Further supporting evidence for the hypothesis is provided by the terrestrially influenced results. In all four correlations shown in Figure 2, the correlation coefficients are lower for the terrestrially influenced samples than the corresponding pristine‐marine samples. In contrast, the absolute concentrations of hyper‐fluorescent PBAP of pristine‐marine and terrestrially influenced samples are similar (as indicated in Section 3.1 and depicted in Figure 2), with respective IQRs spanning 0.37–1.95 L^−1^ and 0.58–2.9 L^−1^. The median value is slightly higher for the terrestrially influenced (1.52 L^−1^) than pristine‐marine (0.87 L^−1^) samples. Altogether, this indicates that the lower correlation values for the terrestrially influenced samples are primarily the result of few observations of much higher PBAP concentrations, which we attribute to additional PBAP sources near coastlines. A similar picture emerges when including the PBAP with weaker fluorescence (3σ threshold) as shown in Figure S5. The conservative, 200 km distance‐from‐land threshold we applied to segregate the measurements (Section 2.5) explains why the terrestrially influenced samples remain similar to the pristine‐marine subset, while the loss of correlation demonstrates the necessity of properly segregating the data set to exclusively isolate those fluorescent particles that are related to SSA production.

### 3.3 Quantification of the Contributions of Fluorescent PBAP to Coarse SSA Concentrations in the Pristine Marine Atmosphere

The moderate correlation observed between (hyper‐)fluorescent PBAP number concentrations and total coarse particle concentrations (Figures 2b and S5b, respectively) for the pristine‐marine samples suggests that the former quantities can be estimated from measurements or calculations of the latter. Histograms of the ratios of hyper‐fluorescent and fluorescent PBAP concentrations to total particle concentrations are shown in Figures S6 and S7. The median values of these ratios are plotted as straight lines in Figures 2b and S5b. These results indicate that for pristine‐marine samples the median percentage contributions of hyper‐fluorescent and fluorescent PBAP to total super‐micrometer SSA concentrations were 0.13% and 1.6%, respectively. For the terrestrially influenced samples, the median percentage contributions of hyper‐fluorescent and fluorescent PBAP to total fluorescent were 0.21% and 2.2%. Although it remains to be seen if similar fractions are obtained in other oceanic regions and during different seasons, these estimates provide a means for estimating super‐micrometer fluorescent PBAP number concentrations from measured or modeled SSA concentrations.

### 3.4 Modulation of Fluorescent PBAP Number Fractions in SSA by Marine Biological Activity

We have demonstrated a clear link between fluorescent PBAP and SSA concentrations in the pristine‐marine atmosphere. According to the previous studies discussed in the Introduction, this link is likely formed by marine microorganisms and DOM that are co‐emitted with sea salt during the SSA production process. Therefore, fluctuations in the abundance of marine biota could potentially modulate the fraction of observed fluorescent aerosols. It is important to note that fixed relationships should not necessarily be expected, given the complex, intermediate aerosol generation and loss processes that link seawater composition with atmospheric aerosol properties. Nevertheless in this section, we qualitatively assess any potential links by examining correlations between seawater composition measurements and the fluorescent aerosol measurements.

Twenty four different types of marine biological and chemical measurements were considered in this analysis. A description of these marine variables is provided in the SI (Section S.3). In short, the marine variables consisted of three distinct classes: (a) number concentrations of different microorganisms obtained from flow cytometry measurements, (b) mass concentrations of different phytoplankton taxa inferred from phytoplankton pigment measurements, and (c) organic matter (OM) measurements which corresponds to DOM (CDOM) and gel‐like POM (TEP and CSP) measurements.

We performed correlation analysis separately for the pristine‐marine and terrestrially influenced groups of measurements in order to isolate the SSA‐related fluorescent PBAP. The number fractions of fluorescent PBAP were considered rather than absolute number concentrations to minimize the risk of falsely identifying associations between the oceanic and atmospheric measurements due to cross‐correlation (e.g., to wind speed, which is an important driver of SSA and marine PBAP production, as shown in Figure 2a, and which might also influence some of the marine variables). In addition, absolute aerosol concentrations are affected by variable atmospheric loss processes, which complicates their use in such a correlation analysis. It is reasonable to assume that similar loss processes occur for similarly sized fluorescent PBAP and non‐fluorescent aerosol particles, and therefore that fluorescent PBAP fractions are much less sensitive to variations in these loss processes.

Number fractions of fluorescent PBAP were calculated by normalizing the coarse fluorescent PBAP number concentrations by the total coarse particle number concentrations simultaneously measured by the WIBS. Marine point samples were extracted from oceanic water with sampling frequencies which varied from 1 to 6 h for different marine variables. To perform the correlation analysis, the results of each marine point sample were simply paired with the overlapping 1 h average of fluorescent PBAP concentration data, justified by limited variation of the latter during 1 h intervals.

**Figure 3:**
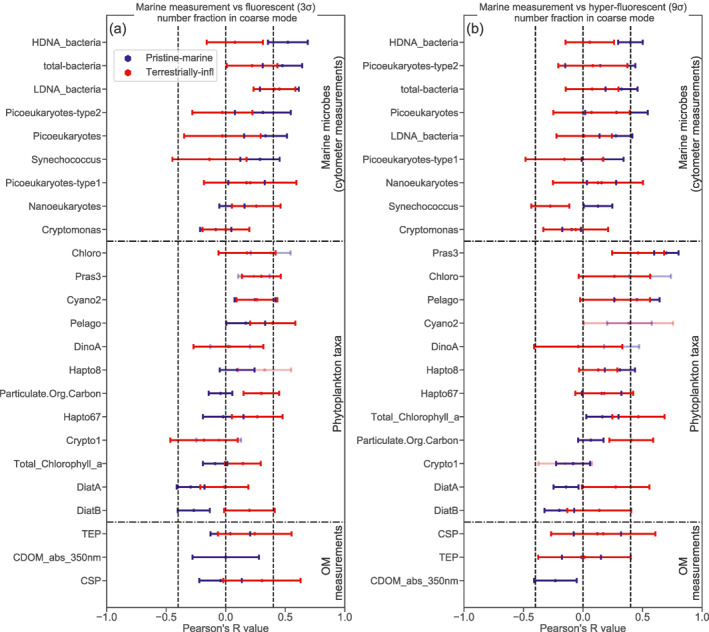
Pearson's correlation coefficients (Pearson's R) from the correlation analysis of marine variables against number fractions of (a) fluorescent primary bioaerosol particles (PBAP) (3σ threshold) and (b) hyper‐fluorescent PBAP (9σ threshold), both relative to total coarse aerosol particle number concentration. The horizontal dash‐dotted lines separate different types of marine measurements namely, cytometer marine microbe number concentration measurements, phytoplankton taxa mass concentration results and OM measurements. The error bars are obtained from the bootstrap analysis. The dimmer colored markers correspond to results of lower statistical significance (p–values > 0.2).

The Pearson coefficients of correlation between coarse fluorescent and hyper‐fluorescent number fractions and the different marine variables are displayed in Figure 3. Corresponding p‐values calculated with a permutation test are shown in Figure S18. The p‐values indicate that the correlation results are statistically significant at the 90% level (i.e., p‐values less than 0.1), with the exception of the Chloro, Cyano2, DinoA, Hapto and Crypto1 results. The results are grouped according to the three marine variable categories (microorganism number concentrations, phytoplankton mass concentrations, and OM measurements). To obtain a measure of uncertainties on the correlation coefficient values a bootstrap analysis was performed for each pair of the analyzed variables (the correlation coefficient calculation was repeated 100 times with random selections containing 60% of all the available data points for each pair of variables). Only those pairs of variables with more than 25 simultaneous data points were considered in this correlation analysis. The corresponding scatter plots for all of the tested variables are displayed in Figures S8–S17.

For both pristine‐marine and terrestrially influenced samples the absolute Pearson's R values associated with the majority of the marine variables were low (−0.4 < R < 0.4). Only a few variables demonstrated more pronounced correlation with the fluorescent (as opposed to hyper‐fluorescent) fraction of coarse particles, with Pearson's R values beyond the −0.4 to 0.4 range. For pristine‐marine results, the variables displaying R > 0.4 were the concentrations of bacteria with high DNA content (HDNA), bacteria with low DNA content (LDNA), total bacteria (sum of the former two types) and picoeukaryotes (small‐sized eukaryotic phytoplankton, typically 1–3 µm). The other types of marine measurements (phytoplankton taxa mass concentrations and DOM related measurements) correlated only weakly with fluorescent PBAP number fractions (*R* < 0.4). This correlation analysis, hence, suggests that the variance of fluorescent particles over the pristine ocean were largely influenced by surface‐ocean bacteria and, to a lesser extent, small phytoplankton.

For the hyper‐fluorescent PBAP number fractions the correlation results are distinct and their rankings are different from those of the fluorescent PBAP number fractions. For the pristine‐marine samples, the prominent correlating marine variables were from the phytoplankton taxa mass concentration results: prasinophytes (Pras3; *R* ≈ 0.69), chlorophytes (Chloro; *R* ≈ 0.55), and pelagophytes (Pelago; *R* ≈ 0.44). In addition to the phytoplankton taxa, concentrations of HDNA bacteria showed moderate correlation (*R* ≈ 0.4), while LDNA bacteria had an R value of 0.27. Similarly to the fluorescent PBAP number fraction results, the DOM related variables only weakly correlate (R values < 0.4) with the hyper‐fluorescent number fractions. The larger contribution of phytoplankton over bacteria to the variance of hyper‐fluorescent PBAP can be expected since phytoplankton cells are larger than bacteria and therefore likely contain more fluorescent components.

The lack of correlation between the OM measurements and the fluorescent and hyper‐fluorescent PBAP fractions does not imply that DOM or gel‐like POM do not contribute to the biologically derived organic matter in SSA. Indeed, transparent expolymeric particles (TEP) and coomasie stainable particles (CSP) were abundant in seawater throughout the entire ACE cruise and therefore, these organic matter components were likely incorporated into SSA particles. The low correlations observed for the OM category in Figure 3 could be due to weak fluorescent emission of the organic compounds comprising DOM and gel‐like POM within the WIBS detection range. Additionally, DOM is expected to be distributed more homogeneously across individual SSA particles compared to insoluble POM. Hence, DOM may be less likely to produce single particles with sufficiently strong fluorescence for detection by the WIBS.

The terrestrially influenced results indicate systematically lower R values for those marine variables that display the highest correlation coefficients with the pristine‐marine samples. Such systematic deterioration of correlation is consistent with the correlation analysis performed in Section 3.2 with the proxy variables for SSA concentrations (Figure 2), which further strengthens the point that the presence of terrestrial aerosols weakens correlations between atmospheric aerosols and marine variables. It is likely that marine biological activity could be enhanced near some of the land masses due to nutrient abundance (Gove et al., 2016), and a few marine variables show correlation coefficients of ∼+0.4 or greater for the air masses in proximity to land. However, such results could be due to cross‐correlations with changes in marine biota near land. Therefore, no attempt is made to further interpret this subset of data. Additionally, it should be noted that terrestrially influenced samples typically possess smaller sample size (the average terrestrially influenced marine samples were ∼25% of the total marine samples) and are statistically less significant than the oceanic samples, as seen from the error bars.

Overall, two main points can be drawn from these correlation results. First (hyper‐)fluorescent aerosol number fractions in the coarse mode correlate best with variables related to marine microorganisms (bacteria and phytoplankton types). This suggests that marine microorganisms are likely incorporated into SSA, and that variations of their concentrations in the ocean modulates the fluorescent fraction of SSA. This strengthens the hypothesis that the observed fluorescent particles are indeed PBAP. Second, the results suggest that those aerosol particles possessing the strongest auto‐fluorescent properties (hyper‐fluorescent particles) correlate to different marine variables than the regularly fluorescing particles. Specifically, the hyper‐fluorescent PBAP fraction correlates more strongly with phytoplankton than bacteria, presumably because phytoplankton are larger and contain more fluorescent material.

Further elaboration is required regarding the different sizes of the marine microbes measured in this study relative to the size detection limits of the WIBS (i.e., aerosol particle diameters from 0.5 to 14 µm). For example, prasinophytes (Pras3) – the mass concentrations of which correlated most strongly with hyper‐fluorescent PBAP number fractions–are amongst the smallest‐sized microalgae. Bacteria, which were among the highest correlating variables with respect to the fluorescent PBAP fractions, are even smaller, with typical sizes in the range of 0.5–1 µm. Conversely, the number concentrations of cryptomonas correlated very weakly with (hyper‐)fluorescent PBAP number fractions. Cryptomonas particles have typical sizes of ∼40 µm, which is generally larger than the other microbes measured in this study, and which may have rendered them undetectable by the WIBS even if they were injected into the atmosphere in SSA. However, such large airborne microbes would display relatively high settling rates and short atmospheric lifetimes, meaning they are less likely to be transported far from their source regions. Therefore, regardless of the limitations of the WIBS measurements, bacteria and small phytoplankton are anyway more likely to contribute substantially to pristine marine PBAP than much larger airborne microbes like cryptomonas.

In conclusion, this correlation analysis suggests that certain types of marine microbes have the potential to modulate the fractions of fluorescent particles in SSA, which is generally consistent with previous studies (e.g., Mayol et al., 2017; Uetake et al., 2020). Further dedicated and targeted measurements are required to confirm if the most highly correlating marine variables observed in this study (concentrations of bacteria and certain phytoplankton types) also have an impact on fluorescent PBAP away from immediate source areas in other oceanic regions and during other seasons.

### 3.5 Classification of Different Fluorescent Particle Types

In this section the fluorescent aerosols are discussed according to the ABC classification scheme of Perring et al. (2015) (Section 2.2 and Table S1). We present classification results for both the pristine‐marine samples and the nine near land events identified in Section 3.1, in order to compare and contrast the fluorescent properties of particles originating from sea spray versus those from the various different terrestrial sources.

**Figure 4:**
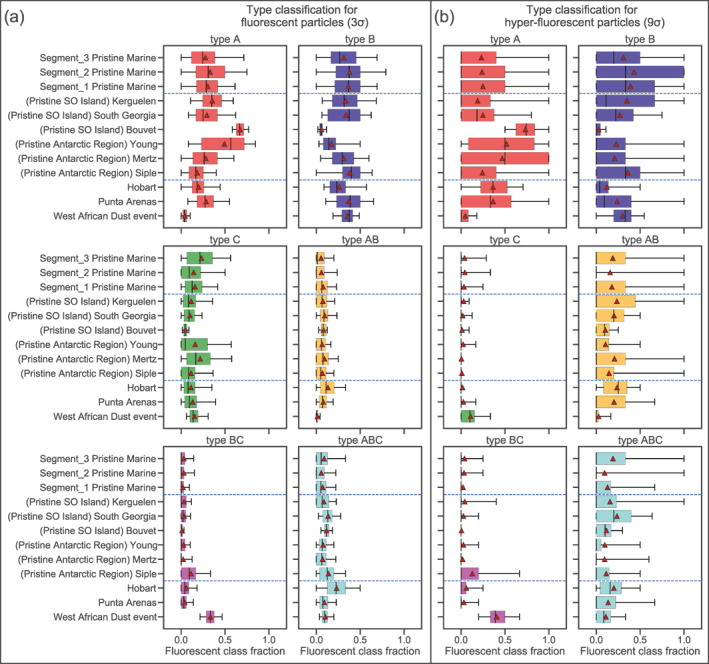
Box and whisker plots of the fraction of fluorescent type for (a) coarse fluorescent (3σ) and (b) coarse hyper‐fluorescent (9σ) particles. The *y* axis represent different names of different test cases for pristine‐marine samples from segment 1 to 3 and near land events. The selected near land events occurred in Hobart‐Tasmania, Mertz Glacier, Young Island, Siple Island, King Edward Point‐South Georgia, and Bouvet Island. In these plots the black whiskers correspond to the 5th and 95th percentile and the boxes are the interquartile ranges. The red dots in the plots represent the mean values.

Figure 4 shows the number fractions of each ABC fluorescence class for the three pristine‐marine cruise segments and the nine near land events. Results are displayed for both the fluorescent and hyper‐fluorescent particles. Since it only made negligible contributions, type AC particles are excluded from Figure 4 for visual clarity.

The most prominent fluorescence classes in the pristine‐marine samples are A, B, AB and ABC. Class C is a prominent class for the particles at the 3σ fluorescence threshold (Figure 4a), but its fractional contribution is substantially reduced for the hyper‐fluorescent (9σ threshold) particles (Figure 4b). The fluorescent particle results indicate that the relative proportions of these classes (mean, median and IQRs) are very similar throughout the cruise segments 1 to 3 (top three rows in each panel). For the hyper‐fluorescent particles, the mean fractions of each class (red triangle markers) are very consistent across segments 1 to 3, while the IQR results for segment 2 are less consistent with the other segments. This could be due to the fact that segment 2 samples were collected further south compared to the other segments, where the presence of sea ice may have resulted in different types of marine microorganisms contributing to the pristine‐marine hyper‐fluorescent PBAP.

The fluorescence class fractions varied more substantially between the nine near land events than they did between the different pristine‐marine cruise segments. For example, the median percentages of type A and B fluorescent particles (3σ threshold) ranged between ∼20%–65% and 5%–50%, respectively, for the near land events, while the corresponding ranges for the pristine‐marine samples were only 25%–30% and 30%–40%, respectively. Such large variability for the near land events can be expected since the composition of fluorescent aerosols and their respective sources might vary substantially between different types of geographical locations.

The fluorescence class fractions for the pristine SO island events (e.g., Kerguelen and South Georgia), and to a lesser extent for the Mertz glacier, are similar to the fluorescence class fractions of the pristine‐marine samples. This might suggest that these near land events were mainly influenced by pristine‐marine aerosol sources. Interestingly, Hobart and Punta Arenas events, which are not regarded as pristine, show fluorescence class fraction compositions which are not significantly distinct from the other pristine near land samples. The only noticeable difference is the higher relative prominence of type ABC and AB particles during the Hobart event.

We also show the fluorescence class fraction West African dust event in Figure 4 (this event was discussed and identified in Section 3.1). The fluorescence class make‐up of the particles measured during this event was distinctly different to those measured during both the pristine‐marine segments and during the other near land events. During the West African event type BC particles were very prominent for the fluorescent samples, while type BC and type C particles were prominent for the hyper‐fluorescent samples. This is an indication that the particles observed during this event possessed distinctly different fluorescent properties compared to the particles that were measured in the SO region. As discussed in Section 3.1, we interpret the fluorescent particles measured during the West Africa event as fluorescing dust aerosols. Therefore, this comparison suggests that long‐range transported dust particles–at least those originating in the Saharan desert and/or those having a similar fluorescence class make‐up as Saharan dust particles–did not contribute substantially to the particles measured over the remote SO during the ACE cruise. This result is in contrast to the study conducted by Crawford et al. (2017) at the Halley VI Research Station in Antarctica in austral summer 2015. They concluded that long‐range transported dust particles, perhaps transported from the southern tip of South America, contributed substantially to the fluorescent particles observed at that Antarctic site.

The observed differences and larger variability in relative fractions of fluorescent particle types for the near land events compared to pristine‐marine samples may also be partly due to the fact that the sample durations of the individual near land events (which only lasted from ∼12 to 48 h) are much shorter than the averages over entire segments for the pristine‐marine samples. To investigate this further a bootstrap analysis was performed separately for each pristine‐marine cruise segment based on 288 randomly selected pristine‐marine data points (which is equivalent to 12 h periods of 5 min averaged data points). These results are presented in Figures S19–S21. They indicate that the subsamples are consistent with the overall results for each segment, which demonstrates that 12 h of data are sufficient to provide statistically robust medians and IQRs for the relative fractions of different fluorescence classes.

A second type of subsampling bootstrap analysis, presented in Figures S22–S24, was based on fixed 24 h' time windows that contained at least 12 h of pristine‐marine data and that were randomly positioned in a segment. They demonstrate some degree of variability in the median and IQR values for subsamples relative to the entire segments, in particular for segment 3. These deviations might reflect inhomogeneity in the types of local marine microorganisms that contribute to the fluorescent particle populations, as well as variations in atmospheric conditions that affect the aerosol sources and sinks on time scales of 24 h, such as passing storms. The variability in fluorescent particle type fractions of terrestrially influenced samples (Figure 4) is larger than that of pristine‐marine subsamples (Figures S22–S24) of comparable duration indicating additional or different fluorescent particle sources.

Size‐resolved ABC classification for pristine‐marine conditions is shown in Figure S25. These results suggest that single type classes (A, B, C) are more dominant in the smaller 2 µm size range, while the fraction of multi‐type classes (AB, BC, ABC) strongly increases for sizes above 2 µm. The increasing contribution of multi‐type classes could be explained by the fact that greater particle volumes are more likely to accommodate sufficient fluorophores of multiple types to exceed the signal thresholds of the corresponding channels.

### 3.6 Further Investigation of the Fluorescent Particle Types–Approximate Humification Index Results

In addition to the ABC WIBS classification scheme, other metrics have been devised to interpret and classify the types of fluorescing compounds and particles that have been observed in various environments. For example, the so‐called humification index has been applied extensively to excitation‐emission spectroscopic measurements of organic matter found in seawater, freshwater, and soils (e.g., Chen et al., 2016; Fu et al., 2015; Zsolnay et al., 1999). In these contexts, the humification index is typically defined as the ratio of emission intensity in the wavelength range from ∼400 – 480 nm to emission intensity in the wavelength range from ∼300 – 350 nm, given an excitation wavelength of 255 nm. The rationale behind this metric is that at this excitation wavelength, protein‐like organic matter tends to display sharper emission profiles at shorter wavelengths, while humic‐like organic matters display broader emission profiles that are shifted to larger wavelength ranges. Therefore, large humification index values (i.e., >∼10) correspond to samples with strong contributions of humified and aromatic organics, while lower humification index values correspond to samples that are either dominated by or contain large contributions from microbially derived protein‐like organic molecules (e.g., Fu et al., 2015).

Unlike emission‐excitation spectroscopy measurements which are typically performed at high spectral resolutions, the WIBS only excites particles at two, discrete excitation wavelengths and then detects the resulting fluorescence signals within two broad emission wavebands. Nevertheless, we can still define an approximate humification index for application to the WIBS measurements, which we denote as the *R*_*B2A*_ ratio to highlight that it is not directly comparable to other humification index results reported in the literature, although they are strongly related. We define this ratio as:

(1) $$R_{B2A}=\frac{FL_{B}}{FL_{A}}\;$$

with *FL*_*A*_ and *FL*_*B*_ being the fluorescence signal amplitude in channel A and B, respectively. That is, the *R*_*B2A*_ ratio is defined as the ratio of fluorescent signal intensity in the wavelength range from 420 to 650 nm to the fluorescent signal intensity in the range from 310 to 400 nm, given an excitation wavelength of 280 nm. One key difference between the *R*_*B2A*_ parameter and the ABC scheme presented in Section 3.4 is that the *R*_*B2A*_ parameter is a continuous variable, while the ABC approach is a binary classification method (i.e., a given signal is either above or below a given channel's threshold). Thus, we calculated *R*_*B2A*_ values for all types of particles, regardless of whether they displayed fluorescent signals above or below the relevant thresholds in channels A and B. However, to prevent measurement noise at low signal levels influencing the results, measured intensities below the 3 (or 9) standard deviation detection thresholds in channels A and B were simply set equal to the mean value of the forced triggering signal for the calculation.

Given that the *R*_*B2A*_ parameter depends on absolute signal intensities, drifts in either or both of the detector channels A and B could contribute to its variability. However, no evidence of substantial drift was observed in the forced trigger data for these two channels over segments 1 to 3, suggesting detector drift didn't contribute substantially to the observed variations in *R*_*B2A*._ Furthermore, given that the absolute signal intensities measured in each channel were not routinely calibrated during the campaign (as is standard operating practice for the instrument, routine calibration is not typically required), we focus here only on the relative comparison between the measurements in this study, and refrain from comparing our *R*_*B2A*_ measurements with humification index results reported in other studies.

**Figure 5:**
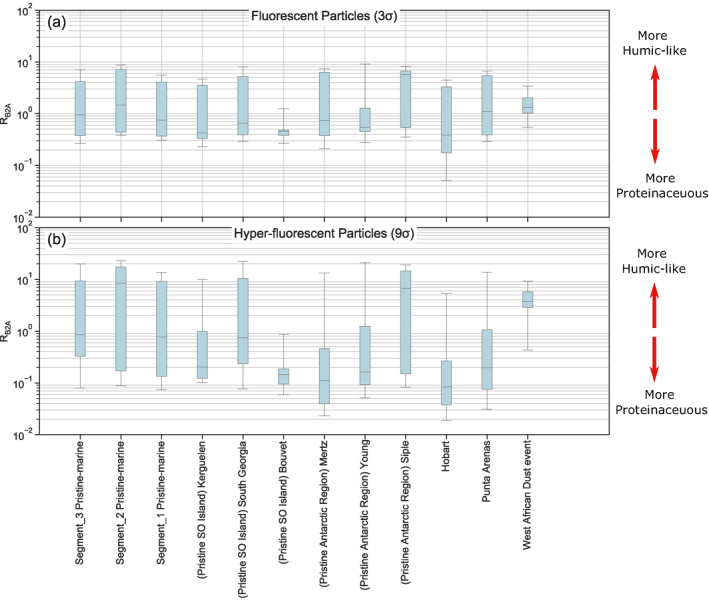
Box and whisker plots of *R*_B2A_ for different test cases for pristine‐marine samples from segment 1 to 3 and near land events. (a) shows results for coarse fluorescent particles while (b) depicts the coarse hyper‐fluorescent particle results. In these plots the black whiskers correspond to the 5th and 95th percentile and the boxes are the interquartile ranges.

Figure 5 shows the box and whisker plots of *R*_*B2A*_ for both fluorescent and hyper‐fluorescent cases. These results show that the IQRs for the pristine‐marine segments 1 to 3 are very similar, while the IQRs for the near land events were more diverse and considerably different from the pristine‐marine results. These results are consistent with the ABC classification results presented in Section 3.4. Both approaches indicate high similarity between pristine‐marine air masses throughout all campaign segments and more variability between individual near land events. This further corroborates sea spray as dominant and quite homogeneous source of fluorescent PBAP in pristine‐marine conditions in the SO.

As evidenced by the broad IQRs displayed in Figure 5, considerable variability in *R*_*B2A*_ was observed for all the events except the Bouvet and West African dust events. This suggests that a broad range of different fluorophore types (both protein‐ and humic‐like) contributed to the fluorescent particles observed during most of the events, whereas specific types of fluorescing matter likely dominated the Bouvet and West African dust events.

The highest median value for *R*_*B2A*_ for the fluorescent aerosol condition is 5.8 which corresponds to the event at Siple Island, indicating that the fluorescent particles in Siple are potentially more humic‐like than the particles observed during the other events. The Siple event was characterized by highly microbially active waters, as well as land‐based penguin colonies and areas of bare soil. Thus, the humic‐like signals may have been caused by high levels of humified and aromatic organics, which may have been produced by increased heterotropy (e.g., as occurs during the decay phase of a phytoplankton bloom), or from water outflows off the Siple coast. The median values for other events are considerably lower and range between 0.4 and 1.5, with the Kerguelen, Bouvet, and Hobart events having the lowest *R*_*B2A*_ (median below 0.5) suggesting that fluorescent particles measured during these events are more protein‐like on average.

The IQRs and median *R*_*B2A*_ results for the hyper‐fluorescent particles differed noticeably from those for the fluorescent particles. In particular, the median *R*_*B2A*_ value for the pristine‐marine segment 2 was substantially higher than the median values during pristine‐marine segments 1 and 3, a difference which was not observed for the fluorescent particles. Indeed, under the hyper‐fluorescent condition, the *R*_*B2A*_ values for pristine‐marine segment 2 are very similar to those measured during the Siple event: median values of 8.4 and 6.8, respectively, the highest median values out of all the events. This indicates higher contributions of humic‐like matter to the most strongly fluorescent particles observed during these two events. In contrast, six of the events (Kerguelen, Bouvet, Mertz, Young, Hobart, and Punta Arenas) displayed median *R*_*B2A*_ values below 0.5 under the hyper‐fluorescent condition (compared to only three events under the fluorescent condition; that is, Kerguelen, Bouvet, and Hobart). This indicates that for these events, the most strongly fluorescent particles contained greater contributions of protein‐like organic matter than the weakly fluorescent particles.

### 3.7 Spatial Variation of Fluorescent PBAP

**Figure 6:**
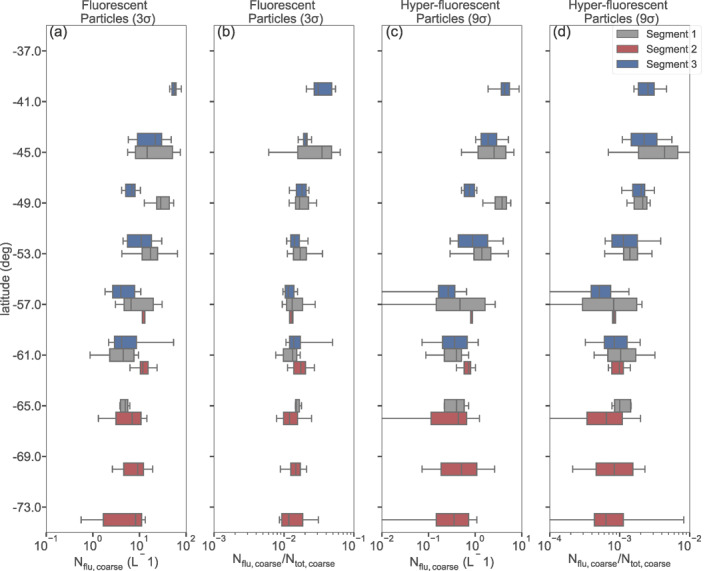
(a) Variation coarse fluorescent number concentration and (b) fraction of coarse fluorescent number concentration to total coarse aerosol number concentration for pristine‐marine samples from different segments of the campaign. (c) Variation of coarse hyper‐fluorescent number concentration and (d) fraction of coarse hyper‐fluorescent number concentration to total coarse aerosol number concentration for pristine‐marine samples. The boxes represent the interquartile ranges and the error bars are the 5th and 95th percentiles.

The ACE cruise covered a latitude range between 34°and 74°S. To assess the latitudinal variability of fluorescent PBAP in pristine‐marine air masses, we grouped the WIBS data in intervals of 4° latitude for each of the three pristine‐marine segments of the cruise. Figures 6a and 6b present box and whisker plots of fluorescent coarse particle number concentrations and fluorescent fractions (relative to total coarse mode number concentrations), and alike for hyper‐fluorescent particles in Figures 6c and 6d.

The median number concentrations ranged from 0.26 to 4.3 L^−1^ and 4 to 56.6 L^−1^ for hyper‐fluorescent (9σ) and fluorescent particles (3σ), respectively. These concentrations correspond to median fluorescent particle percentages in the coarse size range of 0.05%–0.43% for hyper‐fluorescent particles, and 1.1% to 3.4% for fluorescent particles. Overall the particle number concentrations decrease from North to South over the study area. At the same latitude the median values for segment 3 are consistently smaller than for segment 1 (except for hyper‐fluorescent particles near 61°S). This could be interpreted as a seasonal signal (since the segment 1 measurements were performed in January and the segment 3 measurements in March), or a geographical signal, since the segment 1 measurements were performed in the Indian Ocean and the segment 3 measurements were performed in the Atlantic Ocean.

For segment 2 a clear latitudinal trend could not be observed due to the small latitudinal range covered by the segment and the relatively broad IQRs. However, it can be noted that the ranges of median values for the hyper‐fluorescent and fluorescent PBAP (0.35–0.8 L^−1^ and 7 to 11.8 L^−1^, respectively) are not significantly smaller than the corresponding ranges for the most southern parts of the other cruise segments.

Comparing to previous measurements of fluorescent particle number concentrations at high southern latitudes, Crawford et al. (2017) reported average fluorescent number concentrations (based on a 3σ threshold) of 1.9 ± 2.6 L^−1^ at the Halley VI Research Station in Antarctica in austral summer 2015. This corresponded to average percentage of fluorescent particle number fractions of 1.9%. These values are comparable with the corresponding values reported in the present study. However, it should be noted that sampling locations are quite different hindering further detailed interpretations.

### 3.8 Size and Asymmetry Factor (AF) Distributions of Fluorescent Particles

**Figure 7:**
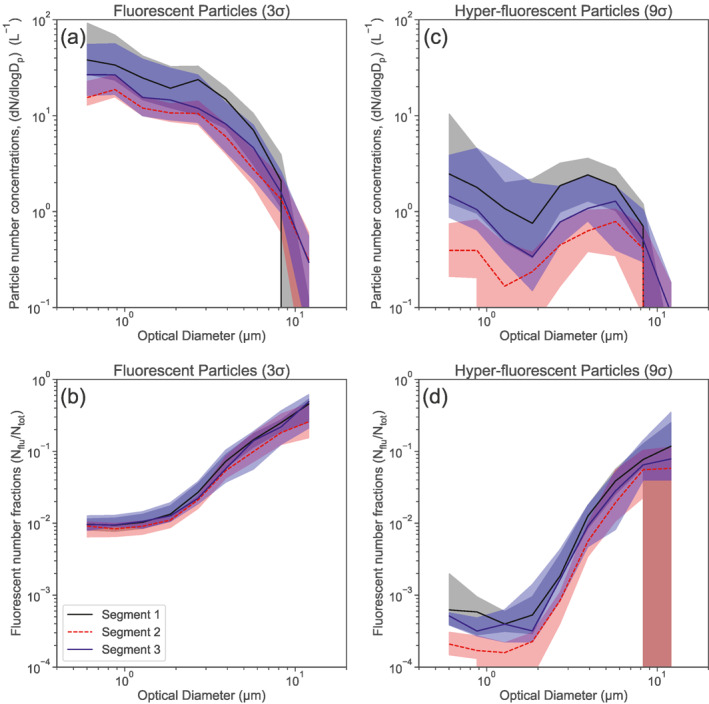
Median optical size distribution for fluorescent (a) and hyper‐fluorescent (b) pristine‐marine samples and size‐resolved fraction of fluorescent to total particles for fluorescent (c) and hyper‐fluorescent particles (d) for the three different segments of the campaign. The shaded color bounds represent the IQR.

Figure 7 shows the median (hyper‐)fluorescent particle size distributions (PSD) along with the corresponding size‐resolved (hyper‐)fluorescent particle fractions for each of the three pristine‐marine segments of the cruise.

The size distribution trends are consistent for segments 1 to 3. Most notably, there is a clear difference between the size‐distributions of the fluorescent and hyper‐fluorescent particles. For the fluorescent particles the size‐resolved concentration decreases continuously as the optical diameter increases. The trend in the hyper‐fluorescent number concentration indicates an initial decrease leading to a minimum at ∼2 µm, followed by a peak number concentration in the range from 5 to 8 µm. This difference in the size distribution shapes suggests that particles larger than ∼2 µm particles emit stronger fluorescence signals compared to smaller particles. The correlation analysis presented in Section 3.4 suggests that phytoplankton are main contributors to hyper‐fluorescent PBAP, whereas bacteria are main contributors to fluorescent PBAP (both assessed for coarse particles with optical diameter > 1 µm). These correlation results are consistent with the size distribution measurements: they suggest that relatively large phytoplankton–a dominant contributor to hyper‐fluorescent PBAP–constitute the mode in the hyper‐fluorescent particles size distributions observed between 5 and 8 µm, while the small phytoplankton, that is, prasinophytes, might be responsible for the signal <2 µm. Second, bacteria, which have generally smaller sizes, have a higher contribution in the fluorescent particle fraction, resulting in higher absolute sub‐micrometer than super‐micrometer fluorescent particle concentrations.

The general trends of the size‐resolved fluorescent particle fractions are similar for both fluorescent and hyper‐fluorescent PBAP (Figures 7b and 7d). The percentage contribution of fluorescent particles in the size range between 0.5 and 2 µm is between 1% and 2%. For particle sizes above 2 µm, the fluorescent percentage increases continuously reaching values of approximately 30%–50% for the largest size bin (14 µm). In the case of the hyper‐fluorescent particles, the corresponding percentages for the size range from 0.5 to 2 µm are between 0.01% and 0.1%, followed by a significant increase to percentages from ∼5% to 13% in the largest size bin. These results indicate that, over the SO, the relative contribution of fluorescent particles to total particle number increases substantially with particle size. This does not necessarily imply an increasing fraction of PBAP with increasing size of SSA particles, as the size dependence of the detected fluorescent particle fraction could be due to larger particles carrying greater quantities of fluorescent compounds.

**Figure 8:**
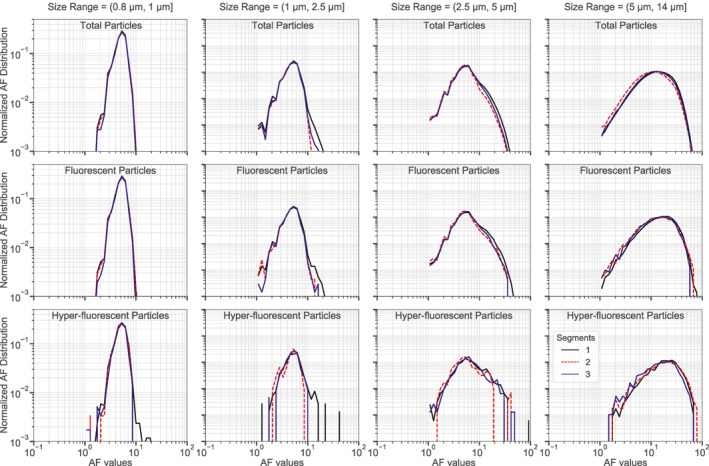
Probability density functions (PDFs) of asymmetry factor (AF) measurements for the pristine‐marine samples from segment 1 (black line), segment 2 (red line) and segment 3 (blue line). Different columns represent different size ranges as indicated in the column titles, while the different rows represent distributions for total aerosol particles (top row), fluorescent particles (3σ) (middle row), and hyper‐fluorescent particles (9σ) (bottom row).

In addition to the size distribution, WIBS also provides information about the shapes of particles through the asymmetry factor (AF) measurements. Toprak & Schnaiter (2013) previously showed that probability density functions (PDF) of spherical particles with different sizes ranging from 1 to 2 µm peak at AF values between ∼8 and 10. Figure 8 shows the normalized PDFs of single particle AF values segregated by size and for total aerosol, fluorescent (3σ) and hyper‐fluorescent (9σ) particles. For particles smaller than 2.5 µm the AF distributions are unimodal with a peak at AF values of ∼5. The AF distribution results are consistent across (hyper‐)fluorescent and total aerosol particles. This indicates that total aerosols and fluorescent particles are essentially spherical in the sub 2.5 µm size range within the limits of the AF resolving power. Given that bacteria likely make an important contribution to the sub‐2.5 µm (hyper‐)fluorescent particle fractions, we speculate that the apparent sphericity of these particles could be due to either bacteria that possess sphere‐like morphologies, or internal mixing of non‐spherical bacteria with DOM components and/or sea salts within individual particles such that the overall particles possess spherical shapes.

The AF PDFs for particles larger than 2.5 µm are also unimodal but with broader distributions compared to the AF PDFs of the sub‐2.5 µm particles. Similarly to the sub‐2.5 µm results, the shapes and widths of the AF PDFs are similar for all particles types (total, fluorescent, and hyper‐fluorescent) in the particle size ranges above 2.5 µm. The modes of the AF PDFs are ∼5 for particles with diameters in the size range from 2.5 to 5 µm, while larger mode values between 10 and 20 are observed for those particles with diameters in the size range from 5 to 14 µm. The larger modes and increased widths for particles with diameters greater than 2.5 µm suggest that these particles are less spherical than the smaller particles. Considering the large (hyper‐)fluorescent fractions for super‐3 µm particles (Figures 7b and 7d), one hypothesis is that the constitutive marine microorganisms of larger PBAP particles are less spherical than the microbes in smaller PBAP particles. On the other hand, the lack of difference between the AF PDFs for the total aerosol particles and for the (hyper‐)fluorescent particles indicates that the morphologies of (hyper‐)fluorescent PBAPs are quite similar to the morphologies of the total aerosol particles, which can include both cubic and more spherically shaped particles (Zieger et al., 2017). That is, the AF results indicate that the biological compounds embedded in PBAP (whether POM or DOM) do not have a major influence on the shapes of SSA particles.

In regards to this discussion on particle shapes it should be noted that the AF measurement applied in the WIBS is not a comprehensive nor sensitive method for investigating particle morphology. For example, a previous laboratory study observed that WIBS‐measured AF increases roughly linearly with increasing particle size for a range of different fluorescing particle types (Savage et al., 2017). These authors were not able to determine if this trend was real or an artifact of the WIBS AF measurement. Therefore, more robust methods (e.g., electron microscopy) should be performed on marine PBAP to further investigate the trends observed in the present study as well as other morphological properties of these aerosols.

## 4 Conclusions

In this study, we presented a comprehensive data set of fluorescent aerosol particle measurements over vast regions of the Southern Ocean (SO). In our analysis we focused on coarse particles (optical diameter > 1 µm) and separated the data into two categories: samples acquired further than 200 km from any land mass (pristine‐marine samples), and samples collected within 200 km from any land mass (terrestrially influenced samples). Furthermore, we used two different instrument fluorescent thresholds (3σ and 9σ) to identify both fluorescent and hyper‐fluorescent particles. The median fluorescent particle number concentrations for the pristine‐marine and terrestrially influenced samples were 11 L^−1^ and 16.6 L^−1^, respectively, while the median hyper‐fluorescent particle number concentration for pristine‐marine and terrestrially influenced samples were 0.87 L^−1^ and 1.47 L^−1^, respectively.

To investigate the relationship between (hyper‐)fluorescent PBAP and SSA a correlation analysis was conducted with four different proxy variables for SSA concentrations (wind speed, total coarse mode particle concentration, Cl‐ and Na + concentrations). Moderately high correlations were observed between pristine‐marine (hyper‐)fluorescent PBAP number concentrations and the SSA proxy variables (e.g., Pearson's R values of 0.76 and 0.61 were obtained between total coarse particle number concentrations and fluorescent and hyper‐fluorescent particle number concentrations, respectively). For all four SSA proxy variables, lower correlation values were obtained for the terrestrially influenced samples relative to the pristine‐marine samples due to existence of outlying measurements that we attribute to potential terrestrial PBAP sources. These results support the hypothesis that SSA is the main source of fluorescent PBAP in pristine marine environments, while also demonstrating the importance of fully isolating pristine‐marine from terrestrially influenced PBAP measurements in order to study them.

Given the high correlation between total and fluorescent particle number concentrations for the pristine‐marine samples, we calculated that fluorescent PBAP represent 1.6% (median value) of the total number of coarse aerosol particles over the pristine SO, while hyper‐fluorescent PBAP represent 0.13% (median value) of the same total. Assuming that in the pristine SO atmosphere SSA is the only significant source of coarse aerosols (on a number basis), these fractions provide a useful means for estimating PBAP number concentrations using measured or modeled SSA number concentrations.

To identify the potential marine sources that modulate fluorescent PBAP concentrations we conducted further correlation analysis with the (hyper‐)fluorescent particle fractions and 30 different marine variables measured in seawater. The results indicated that for pristine‐marine samples, fluorescent particles correlated best with the number concentrations of marine bacteria (Pearson's R = 0.4–0.5), while hyper‐fluorescent particles correlated best with mass concentrations of several different phytoplankton taxa (Pearson's R = 0.4–0.7). In this correlation analysis the terrestrially influenced samples also had systematically lower correlation coefficients compared to the pristine‐marine samples, confirming that the terrestrially influenced samples are likely influenced by non‐marine sources. Overall, the two correlation analyses indicate that the PBAP source flux in the pristine SO is primarily driven by the SSA source flux, with further modulation by seawater concentrations of marine biota such as bacteria and phytoplankton.

To gain insight into the fluorescence characteristics of the measured PBAP, we classified the WIBS measurements using the ABC fluorescence classification scheme. The fluorescence class compositions for the three pristine‐marine segments of the cruise were relatively consistent, which suggests that the sources of pristine marine PBAP were relatively homogenous across all sampled sectors of the SO. In contrast, much more variability was observed between the fluorescence class compositions of nine near land events, which indicates greater diversity in the terrestrial sources of PBAP that contributed to these events. This is not surprising since these events occurred in a wide variety of different environments, including near the Antarctic coast, pristine SO islands, populated continental regions, and even the Saharan desert (the latter occurring during the ship's return voyage back to Europe). The fluorescence class composition of the Saharan dust event was particularly unique (prominent contribution of type BC particles), which suggests that the long‐range transport of dust particles with fluorescence signatures like those of Saharan dust did not contribute substantially to the SO measurements performed during the ACE campaign.

In addition to the ABC classification scheme, we investigated a complementary approach for characterizing aerosol fluorescence properties based on the ratio of fluorescent intensities in channels B and A of the WIBS instrument (termed the *R*_*B2A*_ parameter). The *R*_*B2A*_ results were generally consistent with the ABC classification results: the *R*_*B2A*_ distributions for the three pristine‐marine segments of the cruise were similar while the distributions for the nine near‐land events were much more variable. The highest median *R*_*B2A*_ value was observed for the Siple Island event, which suggests a greater contributions of humic‐like fluorescing matter to the particles comprising this event. The lowest median *R*_*B2A*_ value was observed during the Hobart event, suggesting greater contributions from protein‐like organic matter during this event.

Finally, we summarized the latitudinal variations in (hyper‐)fluorescent particle concentrations and fractions, as well as the (hyper‐)fluorescent particle size and shape parameter (asymmetry factor) distributions. These summaries aim to provide a useful point of comparison for future studies of marine PBAP over the SO as well as other oceanic regions. Of particular interest are the size distribution results, which indicates that while the concentrations of fluorescent particles decreased monotonically from small to large particle diameters, the hyper‐fluorescent particle number size distributions contained a mode between 5 and 7 µm. We suggest that this size distribution mode is associated with the phytoplankton taxa that were observed to correlate highly with the fractions of hyper‐fluorescent PBAP.

## Supporting information

Supporting Information S1

Data Set S1

## Data Availability

The data set used in this study are available in (1) Antoine et al. (2019) available at https://doi.org/10.5281/zenodo.3406983; (2) Chen et al. (2019) available at https://doi.org/10.5281/zenodo.3559982; (3) Landwehr et al. (2020) available at https://doi.org/10.5281/zenodo.3836439; (4) Landwehr, Thurnherr et al. (2020) available at https://doi.org/10.5194/amt-13-3487-2020; (5) Schmale et al. (2019) available at https://doi.org/10.5281/zenodo.2636709; (6) Tatzelt et al. (2020) available at https://doi.org/10.5281/zenodo.3922147; (7) Thomalla et al., (2020) available at https://doi.org/10.5281/zenodo.3859515; (8) Thurnherr et al. (2020) available at https://doi.org/10.5281/zenodo.4031705; In addition, the fluorescent aerosol and gel–like POM measurements have been uploading as supplementary information supporting this article.
