# Supplementary material for: Sources, Occurrence and Characteristics of Fluorescent Biological Aerosol Particles Measured Over the Pristine Southern Ocean
Source: J Geophys Res Atmos. 2021 Jun 9;126(11):e2021JD034811. doi: 10.1029/2021JD034811 (PMC8244095; doi:10.1029/2021JD034811)
Supplement: Supplementary file 1 — Supporting Information S1 [file JGRD-126-e2021JD034811-s002.docx]

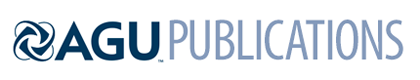


*Journal of Geophysical Research Atmospheres*

Supporting Information for

**Sources, Occurrence and Characteristics of Fluorescent Biological Aerosol Particles Measured over the Pristine Southern Ocean**

Alireza Moallemi^1^, Sebastian Landwehr^1,2^, Charlotte Robinson^3^, Rafel Simó^4^, Marina Zamanillo^4^, Gang Chen^1^, Andrea Baccarini^1,2^, Martin Schnaiter^5,6^, Silvia Henning^7^, Robin L. Modini^1^,*, Martin Gysel-Beer^1^, and Julia Schmale^1,2^,*

1. Laboratory of Atmospheric Chemistry, Paul Scherrer Institute, Villigen PSI, 5232, Switzerland

2. Extreme Environments Research Laboratory, École Polytechnique Fédérale de Lausanne, School of Architecture, Civil and Environmental Engineering, Lausanne, Switzerland

3. Remote Sensing and Satellite Research Group, Curtin University, Kent Street, Bentley 6102 WA, Australia

4. Institut de Ciències del Mar, ICM‐CSIC, Pg Marítim de la Barceloneta 37‐49, 08003 Barcelona, Catalonia, Spain

5. Institut für Meteorologie und Klimaforschung, Karlsruher Institut für Technologie, Karlsruhe, Germany

6. schnaiTEC GmbH, Karlsruhe, Germany

7. Institute for Tropospheric Research, Department of Physics, Leipzig, Germany

Corresponding authors: Robin L. Modini (robin.modini@psi.ch) and Julia Schmale (julia.schmale@epfl.ch)

**Contents of this file**

Text S1 to S9

Figures S1 to S26

Table S1 to S4

**Introduction**

This supporting document contains information on the analysis of wide band integrated bioaerosol senor data, complementary results related to fluorescent and hyper-fluorescent aerosol number concentration, description of marine biological and chemical measurements, and further details regarding ABC fluorescent classification of aerosol particles. Moreover, the document contains scatter plots of (hyper-)fluorescent particle fractions against marine biological and chemical variables. The results demonstrated here were acquired during the Antarctic Circumnavigation Expedition (ACE) in austral summer 2016-2017.

Text S1: Additional description on wide band integrated bioaerosol sensor, sampling inlet and data treatment

## S1.1 The Wide-band Integrated Bioaerosol Sensor (WIBS)

The WIBS inlet flow rate was 2.5 l/min, of which 0.23 l/min is the sample flow and the remaining 2.27 l/min are filtered and used as sheath flow. The WIBS measures the aerosol optical diameter based on elastic light scattering by exposing incoming aerosol particles to a continuous 635 nm diode laser. The light scattered from individual particles is measured in the forward direction by a quadrant photo multiplier tube (PMT) detector, and at a 90˚ angle relative to the laser beam by a second PMT. The aerosol optical diameter in the size range from 0.5 to 14 µm is inferred from the 90˚ side scattering measurements. The forward scattered signals measured by the quadrant detector are used to derive the aerosol asymmetry factor (AF), which is a measure of aerosol morphology. The quadrant detector has four sensors, which measure a portion of the scattered light intensities. The asymmetry factor is obtained by combining these four measured light intensities through the following formula introduced by Gabey et al. (2010) and used in other studies (Savage et al. 2017):

| $AF= \frac{k{(\sum_{i=1}^{n} {(E-E_{i})}^{2})}^{\frac{1}{2}}}{E}$ | Equation S1 |
| --- | --- |

In Eq S1, k is an instrument constant, E is the mean forward scattering signal measured by all the detector sensors, and Ei the scattering signal detected by an individual sensor and n is the number of sensors.

Upon detection of an aerosol particle through the scattering signal, two xenon flash lamps provide UV excitation at wavelengths of 280 and 370 nm sequentially. The fluorescent signals from individual particles are measured in two different channels with bands of 310-400 nm and 420-650 nm. The frequency of the xenon flash lamps and hence the single particle detection frequency is 125 Hz, which implies that a portion of fluorescent particles will not be detected if the aerosol number concentration is above 25’000 L-1. On the other hand, based on the elastic scattering measurements, the WIBS provides the number of missed particle counts between sequential UV source activations. Analysis of the data for different segments revealed that the median of the percentage of missing particle fraction to total aerosol number concentration measured by the WIBS ranged between 5 to 8 %. Figure S1 demonstrate the fraction of missed particle counts (MPC) to the total detected particles particle number (Ntot) for different segments of the campaign. Due to the small portion of missed particles, we did not consider their contribution in this study.


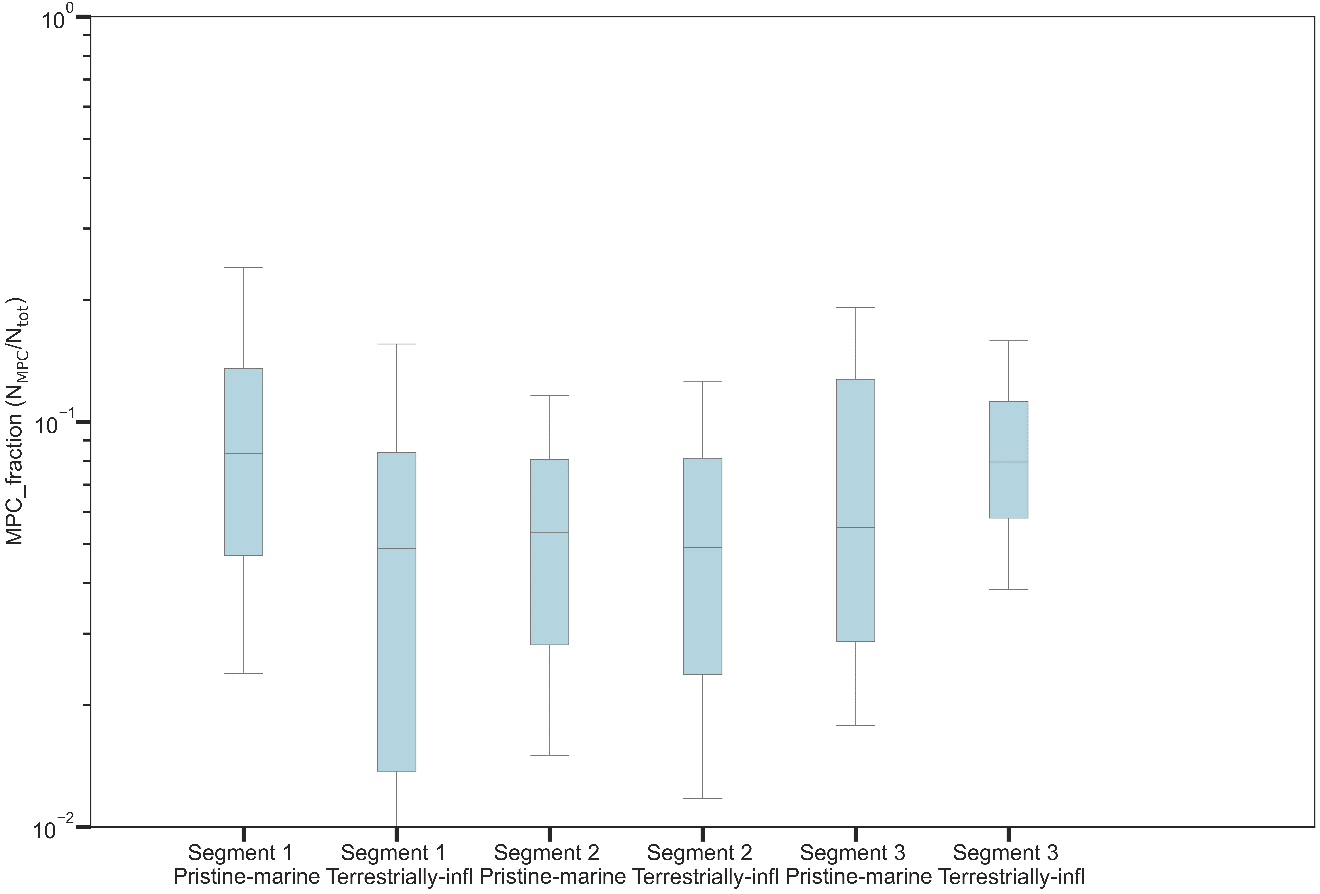


Figure S1. Variation of the fraction of missing particle count to total particle number concentration measured by the WIBS.

The combination of two excitation wavelengths (ExWL) and two emission wavebands (EmWB) provides three different valid fluorescent signal configurations, while one combination is invalid due to interference from the excitation laser. The configuration of the fluorescent channels are:

• Channel 1 (or A): ExWL of 280 nm and EmWB of 310-400 nm

• Channel 2 (or B): ExWL of 280 nm and EmWB of 420-650 nm

• Channel 3 (or C): ExWL of 370 nm and EmWB of 420-650 nm

It should be noted that the ExWL of 280 nm is selected to excite tryptophan while the ExWL of 370 nm is targeted toward excitation of NADH. Moreover, the peaks in the fluorescent signals for tryprophan and NADH occur at EmWB of 310-400 nm and 420-650 nm, respectively.

For this study the method introduced by Perring et al. (2015) was employed for the classification of aerosol particle based on their fluorescent signal. Table S.1 provides the description for all seven fluorescent particle types defined based on the Perring et al. (2015) method.

Table S1. Description of different fluorescence classes following the classification scheme presented by Perring et al. (2015). The AND and NOT in this table correspond to logical ‘and’ and ‘not’, respectively.

| Fluorescence class | Definition of fluorescence class |
| --- | --- |
| A | Fluorescent aerosol detected in channel 1 but NOT in channel 2 and 3 |
| B | Fluorescent aerosol detected in channel 2 but NOT in channel 1 and 3 |
| C | Fluorescent aerosol detected in channel 3 but NOT in channel 1 and 2 |
| AB | Fluorescent aerosol detected in channel 1 AND 2 but NOT in channel 3 |
| AC | Fluorescent aerosol detected in channel 1 AND 3 but NOT in channel 2 |
| BC | Fluorescent aerosol detected in channel 2 AND 3 but NOT in channel 1 |
| ABC | Fluorescent aerosol detected in channel 1 AND 2 AND 3 |

It should be noted that other methods for classifying fluorescent particles are also available. Toprak and Schnaiter (2013) used a slightly different classification method. Their study indicated fluorescent particles detected simultaneously in WIBS channels 1 and 3 could be defined as a robust indicator class for fluorescent bioaeorsol particles (FBAP) with low cross-sensitivity to non-biogenic aerosol. The FBAP class defined in Toprak and Schnaiter (2013) is equivalent to particles identified as AC or ABC based on the classification scheme used in this study.

## S1.2 Sampling inlet

We used a standard Global Atmospheric Watch (GAW) air inlet to direct ambient samples to the instruments. The GAW inlet was 2 m long. Downstream of this main inlet the following inlet configuration made out of conductive tubing was used to deliver sampled air to the WIBS: a 10 cm long line of 25.4 mm outer diameter (21 mm inner diameter), the tubing was then reduced to an 18 mm inner diameter tube through a Swagelok reduction. This tube then extended 50 cm to form a 90 ° bend with as large a radius as possible to avoid particle losses. Using a particle loss calculator (von der Weiden et al., 2009), we calculated that particle losses were on the order of 1 % starting at 7 µm diameter, rising to 10 % at 10 µm diameter. The intake of the main GAW inlet was ~15 m above sea level, which was high enough to avoid direct influence from sea splash against the ship.

The main inlet was heated to keep RH below 40 % according to the GAW recommendations. RH in the inlet line, where it entered the container, was measured with hygrometers in a bypass to not disturb the particle flow. During cruise segments 1 to 3, the RH was less than 30 % and mostly around 20 %. This is because the outside air temperature was much lower than the air temperature inside the container, which was kept constant at 20 °C. During the Saharan dust event, the RH was around 40 % +/- 3% and rose up to 47 % for a couple of hours. This was because of the high temperatures in the tropics, such that the inlet heating could not dry the air sufficiently.

## S1.3 Data removal procedure for potentially contaminated samples by the ship exhaust

For filtering samples suspected of being contaminated by the ship exhaust, aerosol number concentrations (CN, measured by a condensation particle counter with a time resolution of 10 seconds) and ambient CO2 concentrations obtained by a PICARRO (measured at 1 Hz) were used. Then binomial smoothing over 60 data points was applied to both time series. Periods were classified as polluted when the ratio of the 1 minute CN over the smoothed time series was greater than 1.24 or smaller than 0.51, or when the ratio of the 1 minute CO2 signal over the smoothed CO2 time series deviated by 20 %, or when the absolute change between CN at time t and t+1 was larger than 50. In addition to this mask, we used a second filter based on wind direction to further minimize the risk of including ship-exhaust-influenced measurements in the analyzed dataset. Specifically, periods when the wind was blowing from between 90 and 270 ° relative to the ship’s main axis sample (with 0 ° referring to the ships bow being pointed into the wind and 90 ° referring to wind coming from starboard). Approximately 33% of the measurements acquired during segments 1 to 3 were discarded by these two filters. The majority of the removed samples were located near landmasses. Mineral dust particles can also generate measurable fluorescence signals in the WIBS instrument (Savage et al., 2017). However, the results that we present later in Section 3.3 suggest that long-range transported dust aerosols did not contribute substantially to the remote oceanic measurements. Therefore, we assume that all measured particles remaining after application of the ship exhaust filters are PBAP, and we refer to these hereafter as ‘(hyper-) fluorescent PBAP’.

Text S2: APS vs WIBS coarse mode aerosol measurements

Figure S2 shows the scatter plot of hourly averaged integrated number concentrations of total aerosol particles measured by APS and WIBS for particles within the size range of 1 μm to 20 μm, for samples collected in segments 1-3 of ACE and samples collected after segment 3 during the return route from Cape Town to Europe.


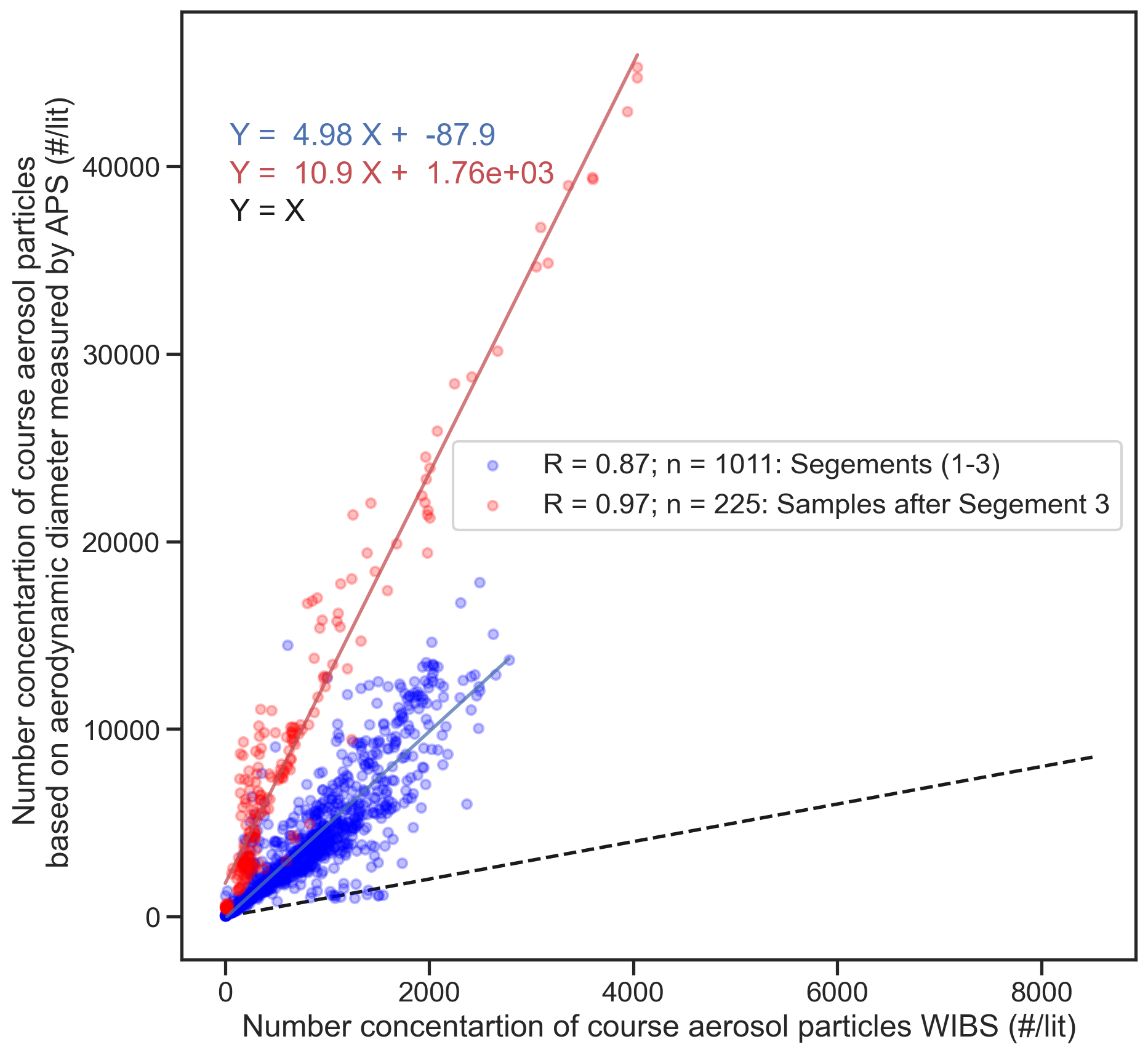


Figure S2. Comparison of particle number concentrations for the diameter range 1 – 20 μm obtained with the WIBS and APS, for samples collected during segments 1-3 and samples collected after segment 3. The correlation coefficient (R) included in the plot corresponds to Pearson’s correlation coefficient.

Text S3: Marine Measurement Description

In this section, marine variables and their analysis methods are presented.

Table S2. Description of marine microbe measurement used in the correlation study against fluorescent aerosol particles.

| Variable | Units | Description | Methods |
| --- | --- | --- | --- |
| HDNA_bacteria-sea-p8 | Cells mL^-1^ | Concentration of high DNA containing bacteria | See section S 4.1 |
| LDNA_bacteria-sea-p8 | Cells mL^-1^ | Concentration of low DNA containing bacteria | See section S 4.1 |
| Total-bacteria-sea | Cells mL^-1^ | Concentration of total bacteria (high & low DNA containing) bacteria | See section S 4.1 |
| Synechococcus-sea-p8 | Cells mL^-1^ | Concentration of *Synechoccocus* sp. Cells | See section S 4.1 |
| Picoeukaryotes-type1-sea-p8 | Cells mL^-1^ | Concentration of picoeukaryote type 1 cells | See section S 4.1 |
| Picoeukaryotes-type2-sea-p8 | Cells mL^-1^ | Concentration of picoeukaryote type 2 cells | See section S 4.1 |
| Nanoeukaryotes-sea-p8 | Cells mL^-1^ | Concentration of nanoeukaryote cells | See section S 4.1 |
| Cryptomonas-sea-p8 | Cells mL^-1^ | Concentration of cryptomonas cells | See section S 4.1 |
| Picoeukaryotes-sea-p8 | Cells mL^-1^ | Concentration of picoeukaryote (type 1 & type 2) cells | See section S 4.1 |

Table S3. Description of marine phytoplankton taxa measurements used in the correlation study against fluorescent aerosol particles

| Variable | Units | Description | Methods |
| --- | --- | --- | --- |
| Particulate.Org.Carbon-p1 | $\mu$M | Particulate organic carbon concentration | See section S 4.2 |
| Total_Chlorophyll_a_merged-p1 | $\mu$g L^-1^ | Total chlorophyll-a concentration | See section S 4.3 |
| Chloro | $\mu$g L^-1^ | chlorophyte contribution to chlorophyll biomass | See section S 4.4 |
| Crypto1 | $\mu$g L^-1^ | Cryptophyte contribution to chlorophyll biomass | See section S 4.4 |
| Cyano2 | $\mu$g L^-1^ | Cyanobacteria type 2 contribution to chlorophyll biomas | See section S 4.4 |
| DiatA | $\mu$g L^-1^ | Diatom type contribution to chlorophyll biomas | See section S 4.4 |
| DiatB | $\mu$g L^-1^ | Diatom type 2 contribution to chlorophyll biomas | See section S 4.4 |
| DinoA | $\mu$g L^-1^ | Dinoflagellate type 1 contribution to chlorophyll biomas | See section S 4.4 |
| Hapto8 | $\mu$g L^-1^ | Haptophyte type 8 contribution to chlorophyll biomas | See section S 4.4 |
| Haptophyte67 | $\mu$g L^-1^ | Haptophyte type 6&7 contribution to chlorophyll biomas | See section S 4.4 |
| Pras3 | $\mu$g L^-1^ | Prasinophyte type 3 contribution to chlorophyll biomas | See section S 4.4 |
| Pelago | $\mu$g L^-1^ | Pelagophyte contribution to chlorophyll biomas | See section S 4.4 |

Table S4. Description of other marine organic measurements.

| **Dissolved Compounds** | | | |
| --- | --- | --- | --- |
| Variable | Units | Description | Methods |
| CDOM_abs_350nm | m^-1^ | Colored dissolved organic material (CDOM) absorption at 350 nm | See section S 4.5 |
| TEP | µg XG eq L^-1^ | Transparent Exopolymeric Particles | See section S 4.6 |
| CSP | µg BSA eq L^-1^ | Coomasie Stainable Particles | See section S 4.6 |

Text S4: Description of methods used for marine measurement

## S4.1 Marine microbe number concentration measurements

Number concentration of bacteria and pico-, nano- and microalgae in sea water were measured through cytometry. After extraction, sea water samples were aliquoted in cryovials. For each samples 4.5 ml duplicates and 1.8 ml replicate were collected. The samples were treated by 1% paraformaldehyde plus 0.05% glutaraldehyde and kept at – 80 ˚C until analysis on land. After thawing, samples were analysed with a PARTEC Cube 8 flow cytometer equipped with a laser emitting at 488 nm. Heterotrophic bacteria were counted by their signature in a plot of side scatter versus green fluorescence after being stained with 10 µM of SYBRGreen I. In separate runs of unstained samples, pico- and nano-phytoplankton and cryptomonas cells were identified and enumerated on the basis of the differences in autofluorescence and light scattering characteristics.

## S4.2 Particulate organic carbon concentration measurements

Particulate organic carbon was measured by extracting 2000 ml of sea water samples and filtering them using 25 mm combusted 0.3 µm Glass Fibre filters (GF-75; Sterlitech). After sample extraction, the filter papers were kept in combusted tinfoil and cooled down to -80 °C. The filters were analyzed in University of Cape Town using an elemental analyser-isotope ratio mass spectrometer (Walton and Thomas, 2018). The particulate organic carbon data could be found in (Thomalla et al., 2020).

## S4.3 Merged total chlorophyll-a

Absolute concentrations of total chlorophyll-a pigment concentration were derived via high performance liquid chromatography (HPLC, Antoine et al., 2019) at locations roughly every 6-12 hours. Measurements of particulate absorption were collected at a higher resolution, roughly every 3-6 hours. Using matched samples of HPLC derived total chlorophyll-a and particulate absorption, the absorption line height method of Roesler & Barnard (2013) for determining total chlorophyll-a concentration was calibrated and applied to the whole particulate absorption dataset in order to increase the resolution of the total chlorophyll-a concentration estimations

## S4.4 Phytoplankton CHEMTAX

The data on phytoplankton taxonomy groups and their contributions were obtained from the pigment concentration measurements (Antoine et al., 2019) and by using CHEMTAX v1.95 chemical taxonomy software (Mackey et al., 1996). The quantified taxonomy groups in this studies are: Chlorophytes type 1, cryptophytes type 2, diatoms type 1, diatoms type 2, dinoflagellates type 1, haptophytes type 8, haptophytes types 6 + 7, prasinophytes, and pelagophytes (Higgins at al., 2011).

Before conducting CHEMTAX analysis, the data was pre-processed and clustered. The data was standardized was based on mean subtracted and divided by standard deviation. Prior to clustering the data, a dissimilarity matrix was computed based Manhattan’s distances. Hierarchical clustering (Ward’s method) was used for clustering analysis and the Elbow, silouette and gap tests indicated the existence of 5 clusters. The CHEMTAX analysis was conducted on the clustered data. Initially, to obtain the matrices of optimized pigment rations, 60 analysis runs were performed on each individual clustered. This was followed by a final 20 analysis runs on the data to calculate the taxonomic abundance. In this study the initial pigment ratios were gathered from Rodriguez et al. (2002) (2002), Zapata et al. (2004), Cook et al. (2011) and Higgins at al. (2011), Cassar et al. (2015), Nunes et al. (2019).

## S4.5 Coloured dissolved organic matter (CDOM) concentration measurements

Coloured dissolved organic matter is a component dissolved organic matter (DOM) in seaweter which strongly absorbs light in the ultraviolet wavelengths. CDOM is typically strongly correlated with DOM and could be used as a proxy for DOM.

The absorption spectra of the CDOM from the collected sample were measured onboard with a UV-spectrometer, and the data included in this analysis corresponds to the absorption of CDOM at wavelength of 350 nm. Further information can be found in the cruise report (Walton and Thomas, 2018).

## S4.6 Transparent Exopolymeric Particles (TEPs) and Coomasie Stainable Particles (CSPs) measurements

Transparent exopolymeric particles (TEP) and coomassie-blue stainable particles (CSP) are gel-like compounds that are rich in polysaccharide and protein, respectively. Seawater samples (150-300 ml) were filtered through 25 mm diameter 0.4 μm pore size polycarbonate filters. For TEP analysis the filters were stained with 500 μL of Alcian blue solution (0.02 %, pH 2.5) for 5 s, rinsed with Milli-Q water and stored frozen. For CSP analysis, the filters were stained with 700 μL of a working Coomassie Brilliant Blue (CBB-G 250) solution (0.04 %, pH 7.4) for 30 s, rinsed with Milli-Q water and stored frozen. For each batch of TEP and CSP samples duplicate blank filters which were not stained were collected. Measurements of TEP and CSP were conducted in land laboratories. For TEP all the samples and blank filters were treated in 5 ml of 80% sulfuric acid and shaken intermittently for 3 h. The measurement was conducted by a spectrophotometre at 787 nm (Varian Cary spectrophotometer). For CSP all the samples and blank filters were treated in 4 mL of extraction solution (3 % SDS in 50 % isopropyl alcohol) and sonicated in a water bath at 37º C for 2 hours. The CSP measurement was conducted y a spectrophotometre at 615 nm (Shimadzu UV–Vis UV120). The Alcian blue dye solution calibration was performed using a standard solution of Xanthan Gum (XG). The CBB dye solution calibration was performed using bovine serum albumin standard (BSA).

# Text S5: Correlation analysis of SSA proxies vs fluorescent aerosols at different land proximity values

To find a reasonable proximity to land distance, the Pearson’s R values of the different proxy variables against fluorescent and hyper-fluorescent coarse particles were obtained as a function of the distance to land. Figures S3 and S4 show the results for fluorescent and hyper-fluorescent particles, respectively.


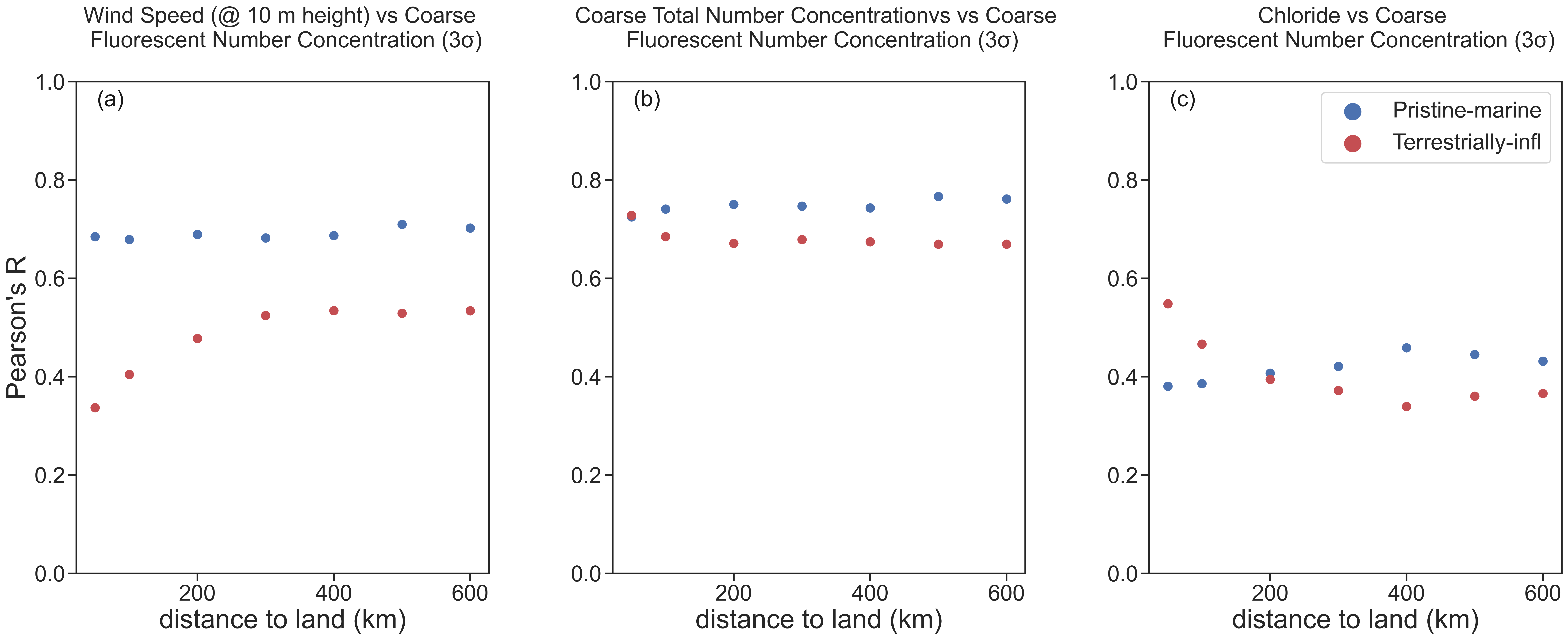


Figure S3. Pearson’s R values for pristine-marine and terrestrially-influenced air masses of fluorescent particles for different land proximity values.


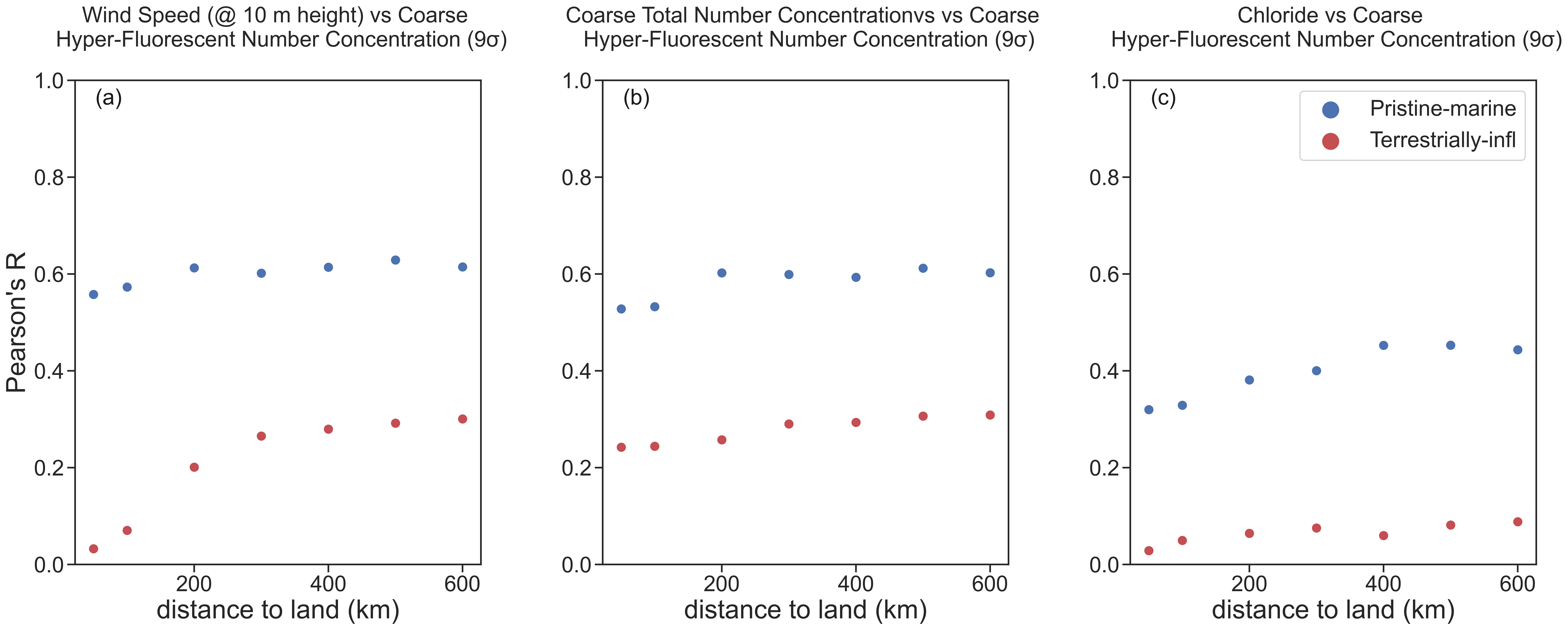


Figure S4. Pearson’s R values for pristine-marine and terrestrially-influenced air masses of hyper-fluorescent particles for different land proximity values.

# Text S5: Scatterplots of fluorescent particle (3σ) concentrations against the four proxy variables for SSA concentrations

The scatter plots for fluorescent coarse particles vs SSA proxies are presented in Figure S5.





Figure S5. Scatter plots of pristine-marine and terrestrially-influenced air masses of fluorescent particles vs SSA proxies for the combined segment 1 to segment 3 results. The red and blue shades correspond to the interquartile ranges (IQR) of the measurements that were calculated by binning the dataset into ten equidistant logarithmic bins.

# Text S6: Distribution of the number concentration fraction of fluorescent PBAPs to coarse SSA number concentrations

The histograms of the fraction of (hyper-)fluorescent number concentrations to total coarse aerosol particle number concentrations based on hourly averaged data are shown in Figures S6 and S7.


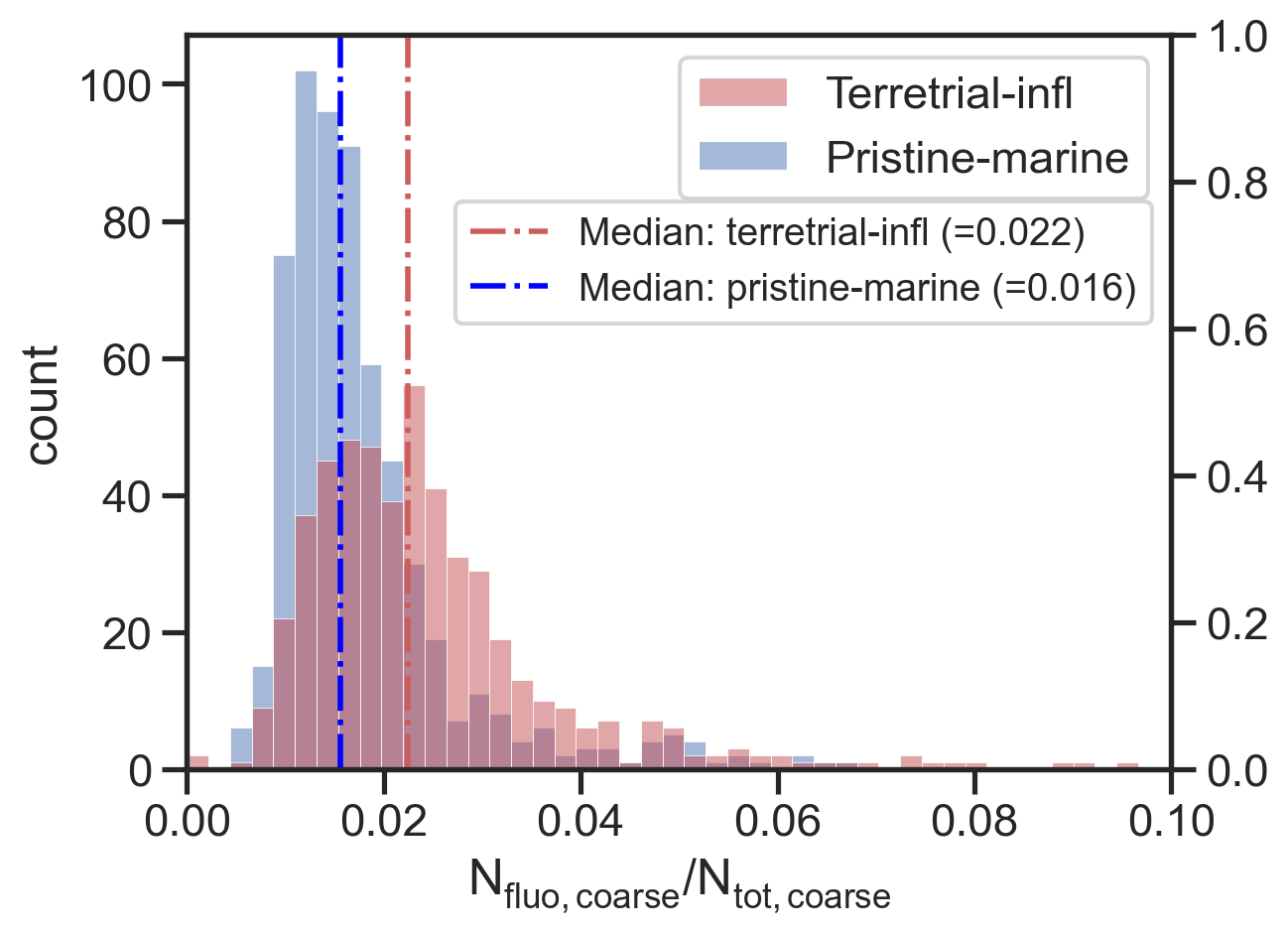


Figure S6. Distribution of number fraction of fluorescent PBAP to total coarse particle number concentration.


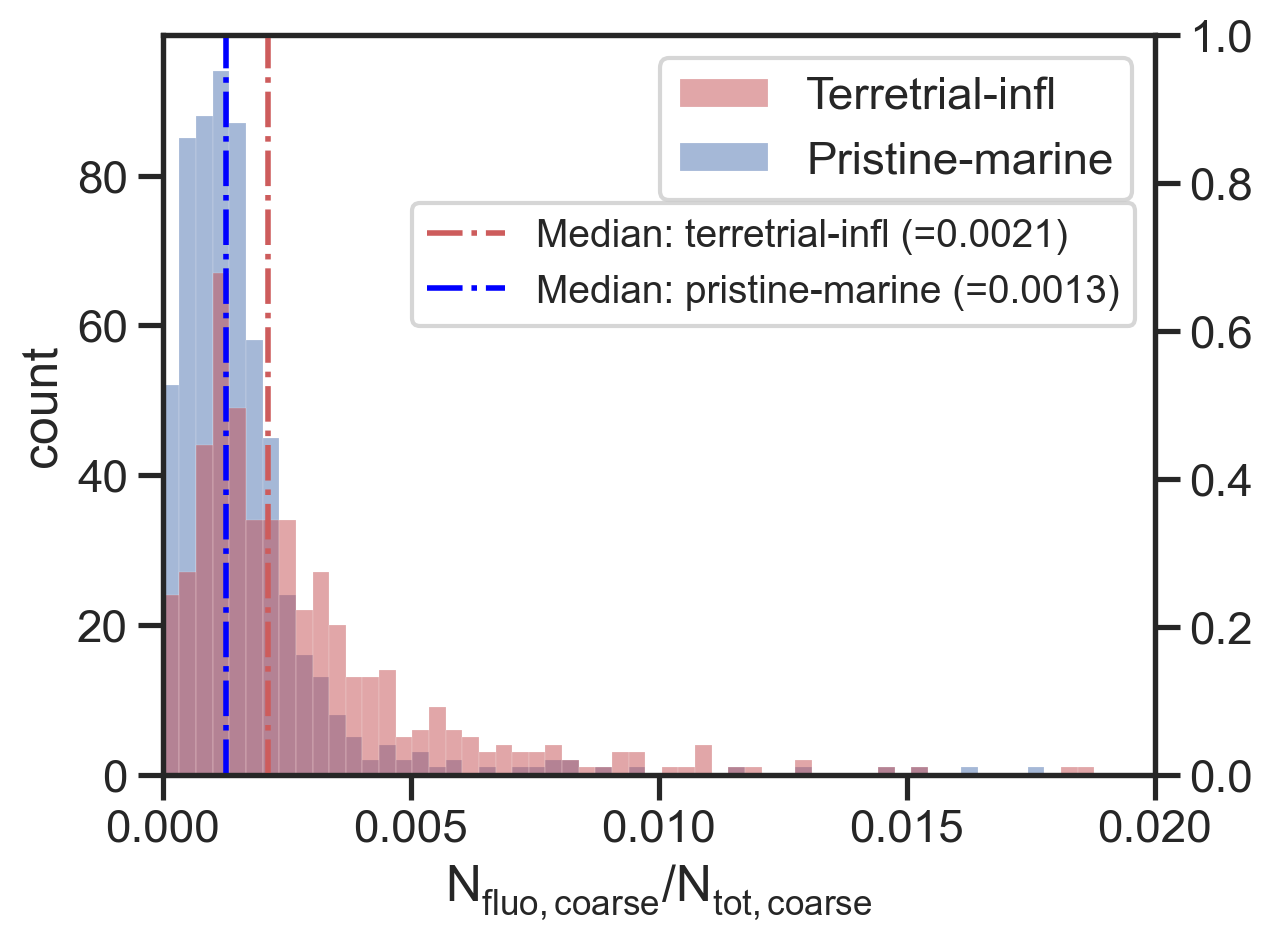


Figure S7. Distribution of number fraction of hyper-fluorescent PBAP to total coarse particle number concentration.

# Text S7: Scatter plots of different marine variables against normalized fluorescent number concentration

## S7.1 Fluorescent particle number concentration fraction vs phytoplankton taxa

Figures S8 and S9 show the results of the fraction of coarse fluorescent particle number concentrations to total coarse particles against marine measurements associated with phytoplankton taxa. All the fit lines in the plots demonstrated in section S7 correspond to linear regressions that were applied on the datasets. The Pearson’s R values are also included.





Figure S8. Scatter plot of fraction of coarse fluorescent particle number concentrations to total coarse particles vs. different phytoplankton taxa measurements.


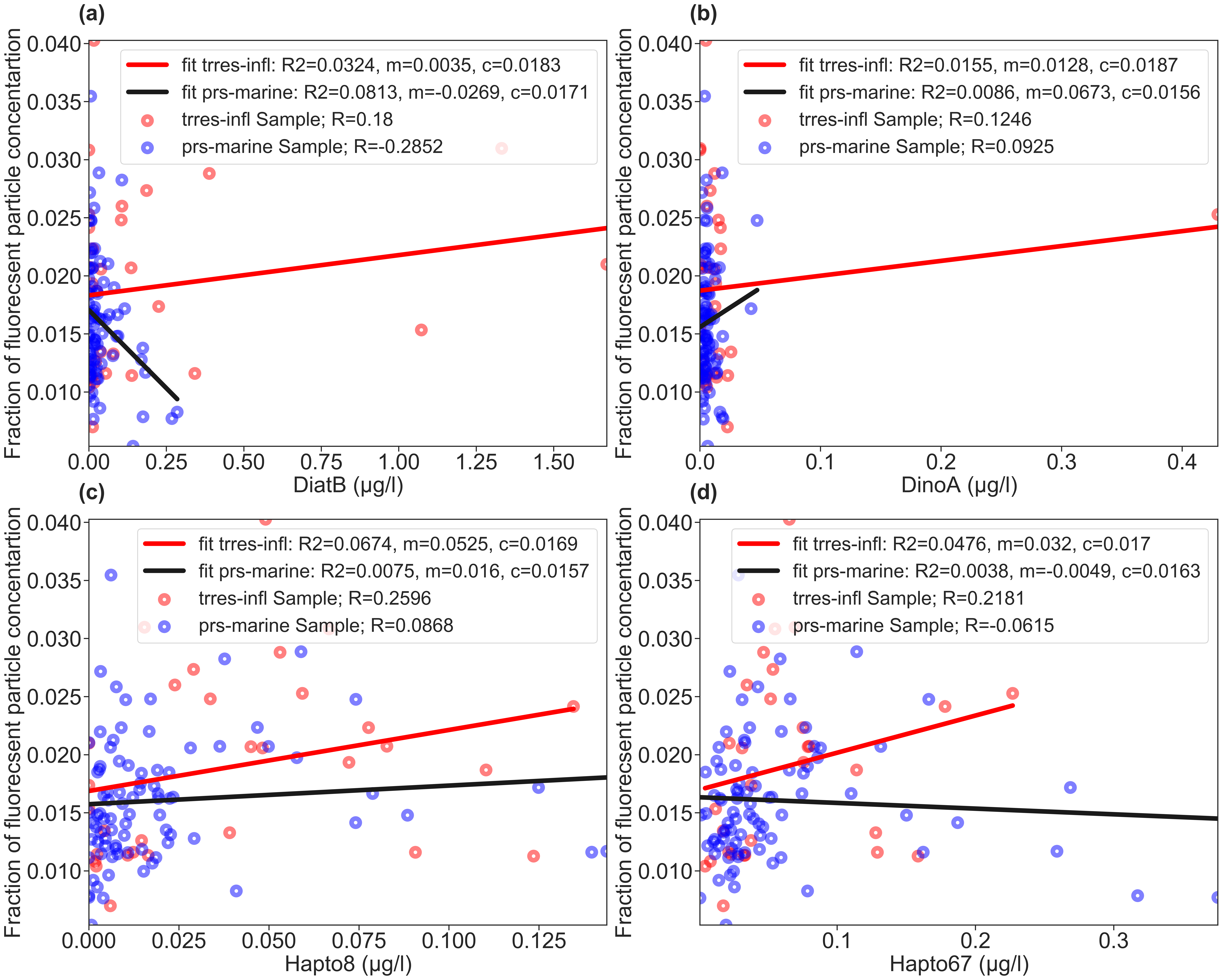


Figure S9. Scatter plot of fraction of coarse fluorescent particle number concentrations to total coarse particles vs. different phytoplankton taxa measurements

## S7.2 Fluorescent particle number concentration fraction vs marine microbe measurements

Figures S10 and S11 show the results of the fraction of coarse fluorescent particle number concentrations to total coarse particles against marine measurements associated with marine microbe measurements. Fits are analogue to S 9.1.





Figure S10. Scatter plot of fraction of coarse fluorescent particle number concentrations to total coarse particles vs. different marine microbe measurements


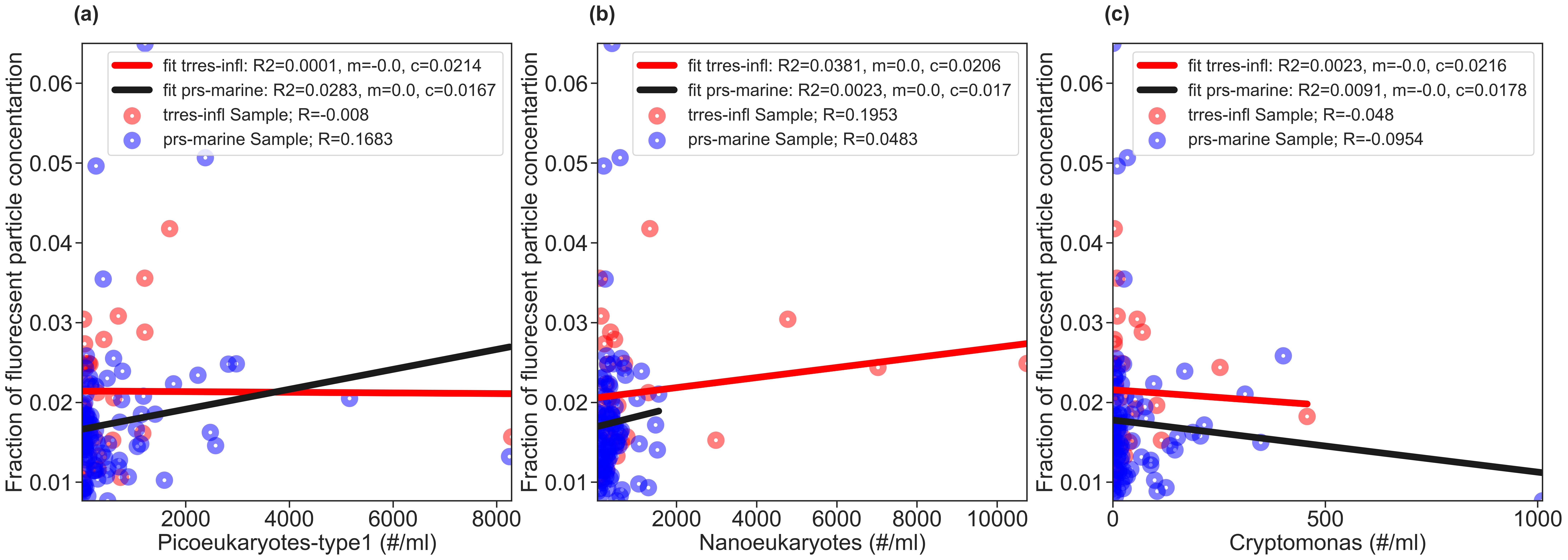


Figure S11. Scatter plot of fraction of coarse fluorescent particle number concentrations to total coarse particles vs. different marine microbe measurements

## S7.3 Fluorescent particle number concentration fraction vs organic matter (OM) measurements

Figure S12 shows the results of fraction of coarse fluorescent particle number concentrations to total coarse particles against OM measurements. Fits are analogue to S 9.1.


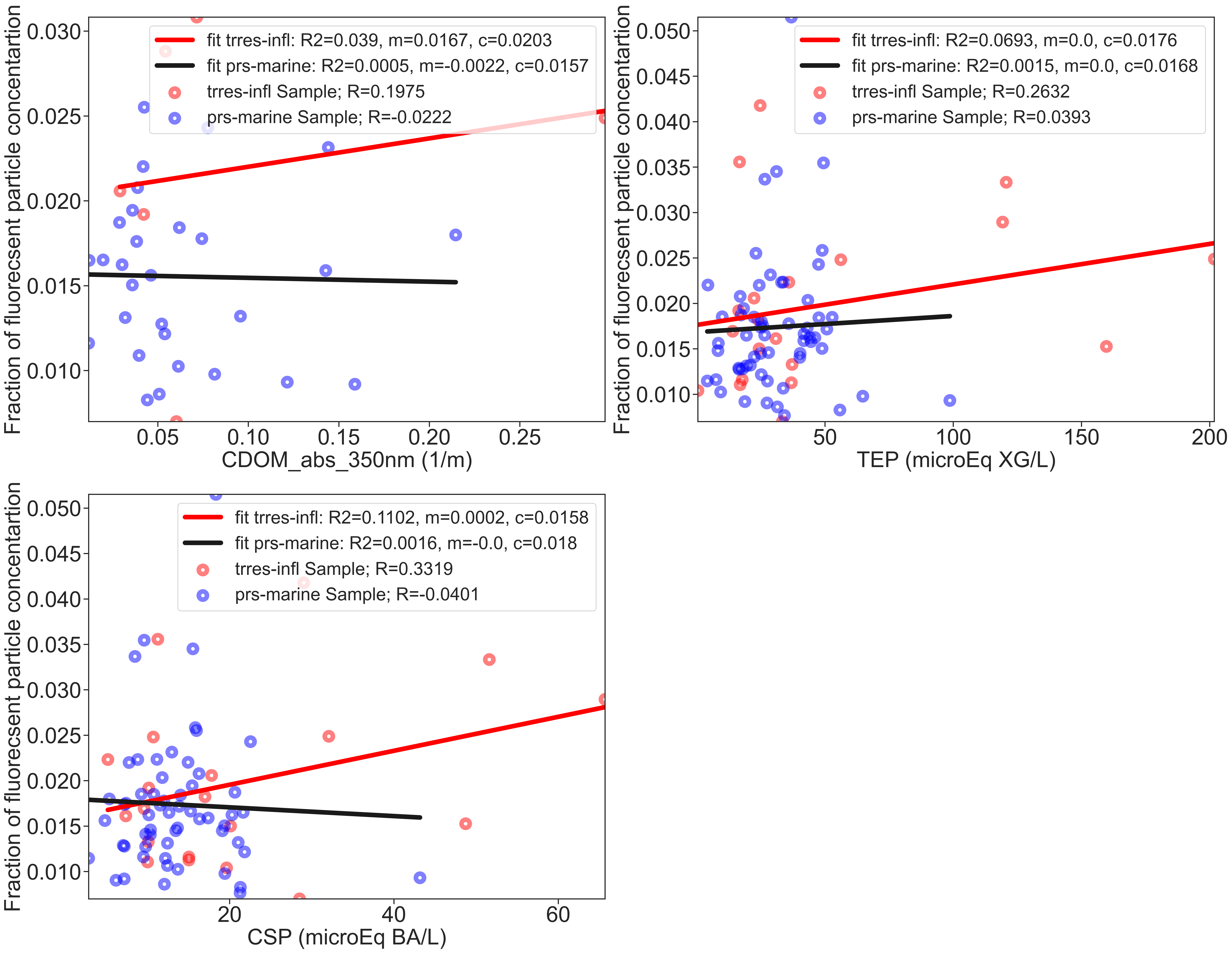


Figure S12. Scatter plot of fraction of coarse fluorescent particle number concentrations to total coarse particles vs. OM measurements.

## S7.4 Hyper-fluorescent particle number concentration fraction vs phytoplankton taxa

Figures S13 and S14 show the scatter results of fraction of coarse hyper-fluorescent particle number concentrations to total coarse particles against marine measurements associated with phytoplankton taxa.





Figure S13. Scatter plot of fraction of coarse hyper-fluorescent particle number concentrations to total coarse particles vs. different phytoplankton taxa measurements


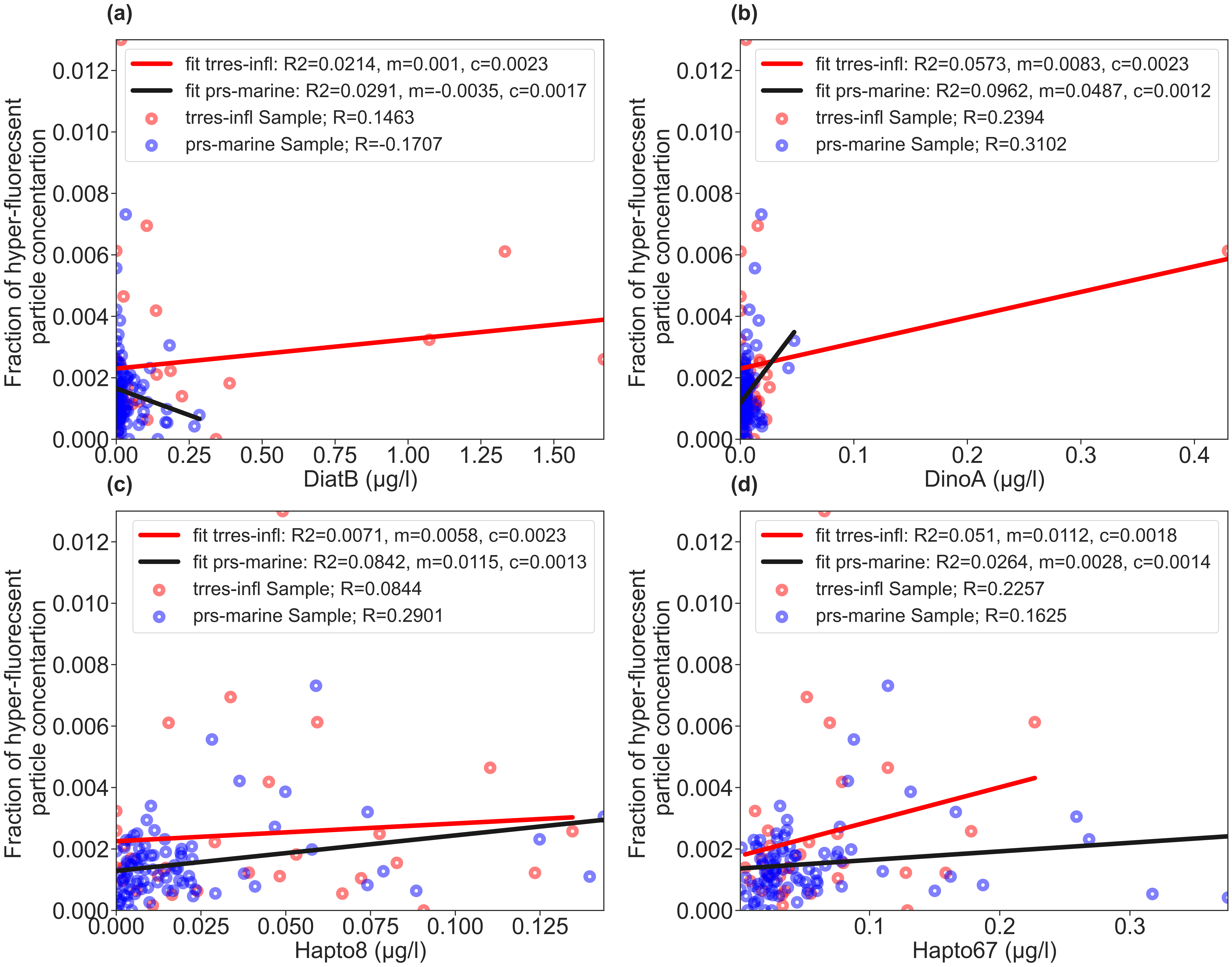


Figure S14. Scatter plot of fraction of coarse hyper-fluorescent particle number concentrations to total coarse particles vs. different phytoplankton taxa measurements

## S7.5 Hyper-fluorescent particle number concentration fraction vs marine microbe measurements

Figures S15 and S16 show the scatter results of fraction of coarse hyper-fluorescent particle number concentrations to total coarse particles against marine measurements associated with marine microbe measurements.





Figure S15. Scatter plot of fraction of coarse hyper-fluorescent particle number concentrations to total coarse particles vs. different marine microbe measurements


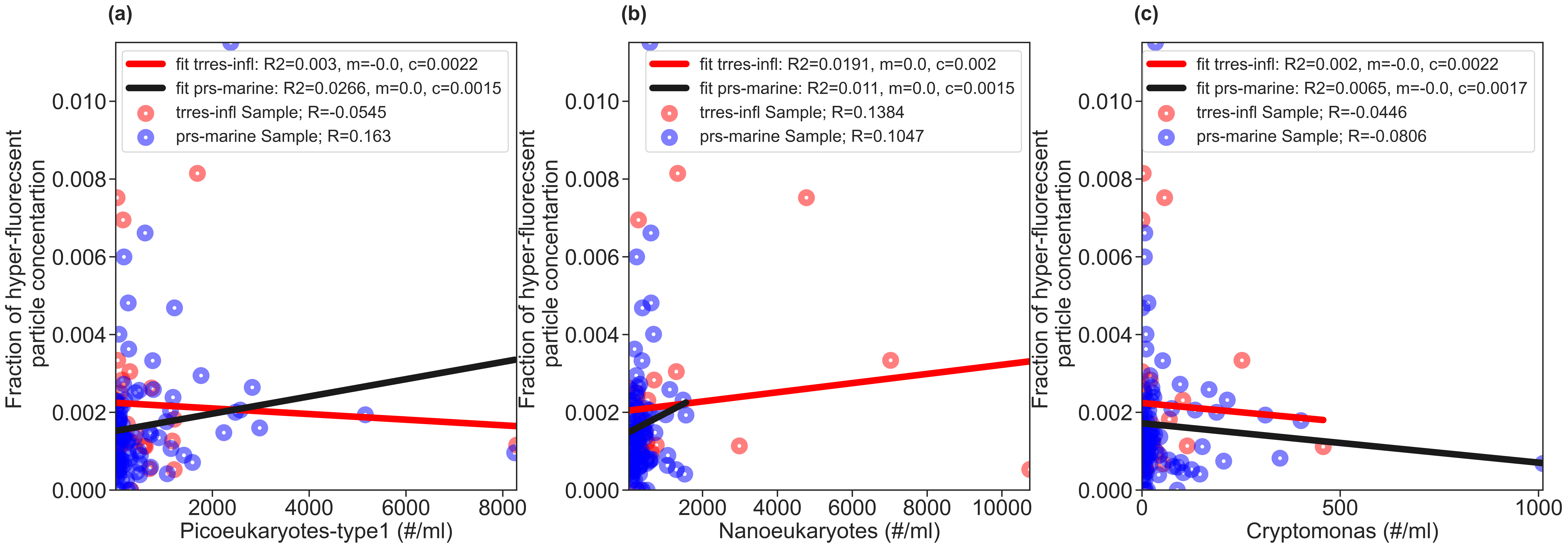


Figure S16. Scatter plot of fraction of coarse hyper-fluorescent particle number concentrations to total coarse particles vs. different marine microbe measurements

## S7.6 Hyper-fluorescent particle number concentration fraction vs OM measurements

Figure S17 shows the results of fraction of coarse fluorescent particle number concentrations to total coarse particles against OM measurements. Fits are analogue to S 9.1.ss


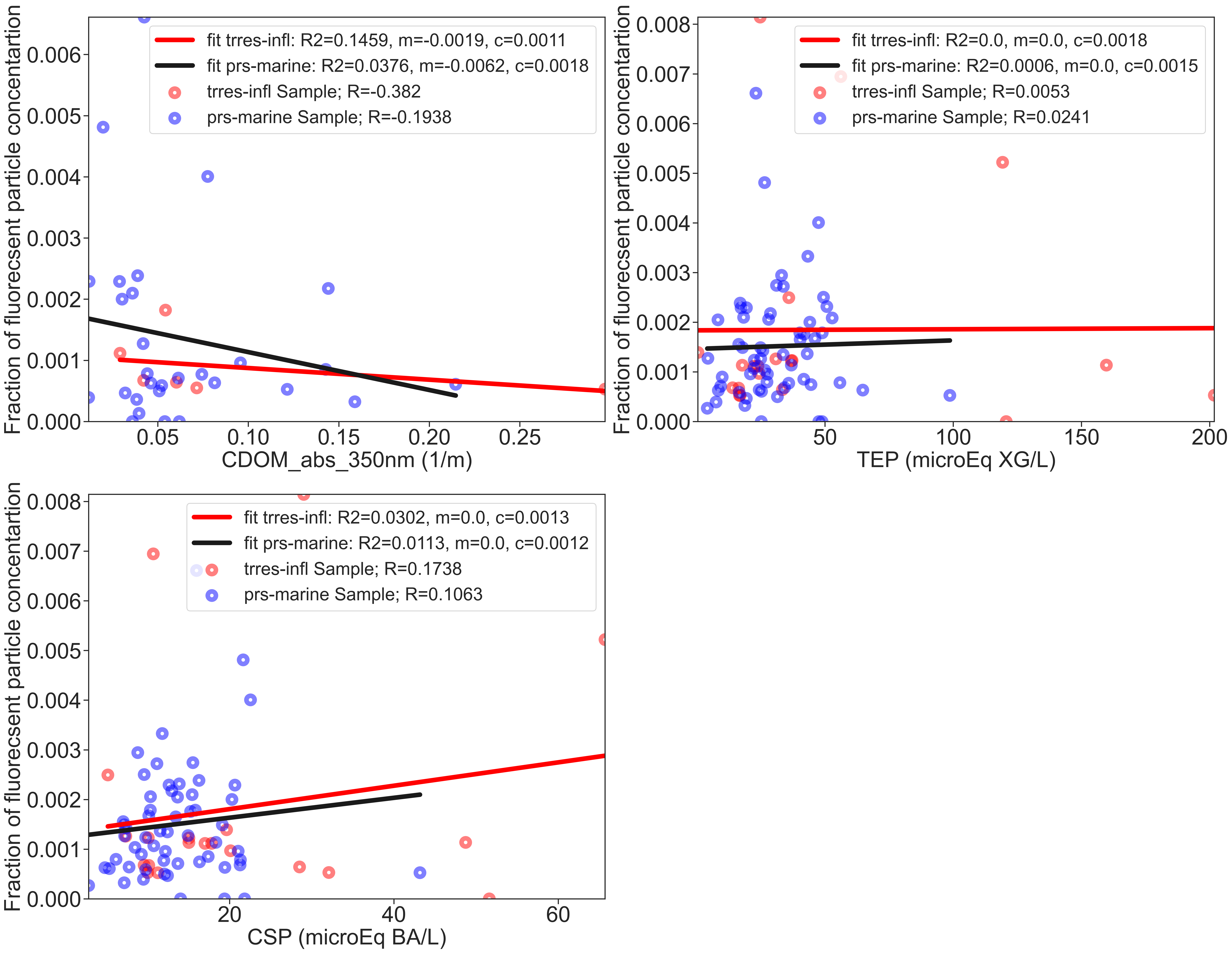


Figure S17. Scatter plot of fraction of coarse hyper-fluorescent particle number concentrations to total coarse particles vs. OM measurements

# Text S8: p value results of marine measurement

The p values for the marine varibales used in the correlation study against (hyper-fluorescent particles) are demonstrated in Figure S18.


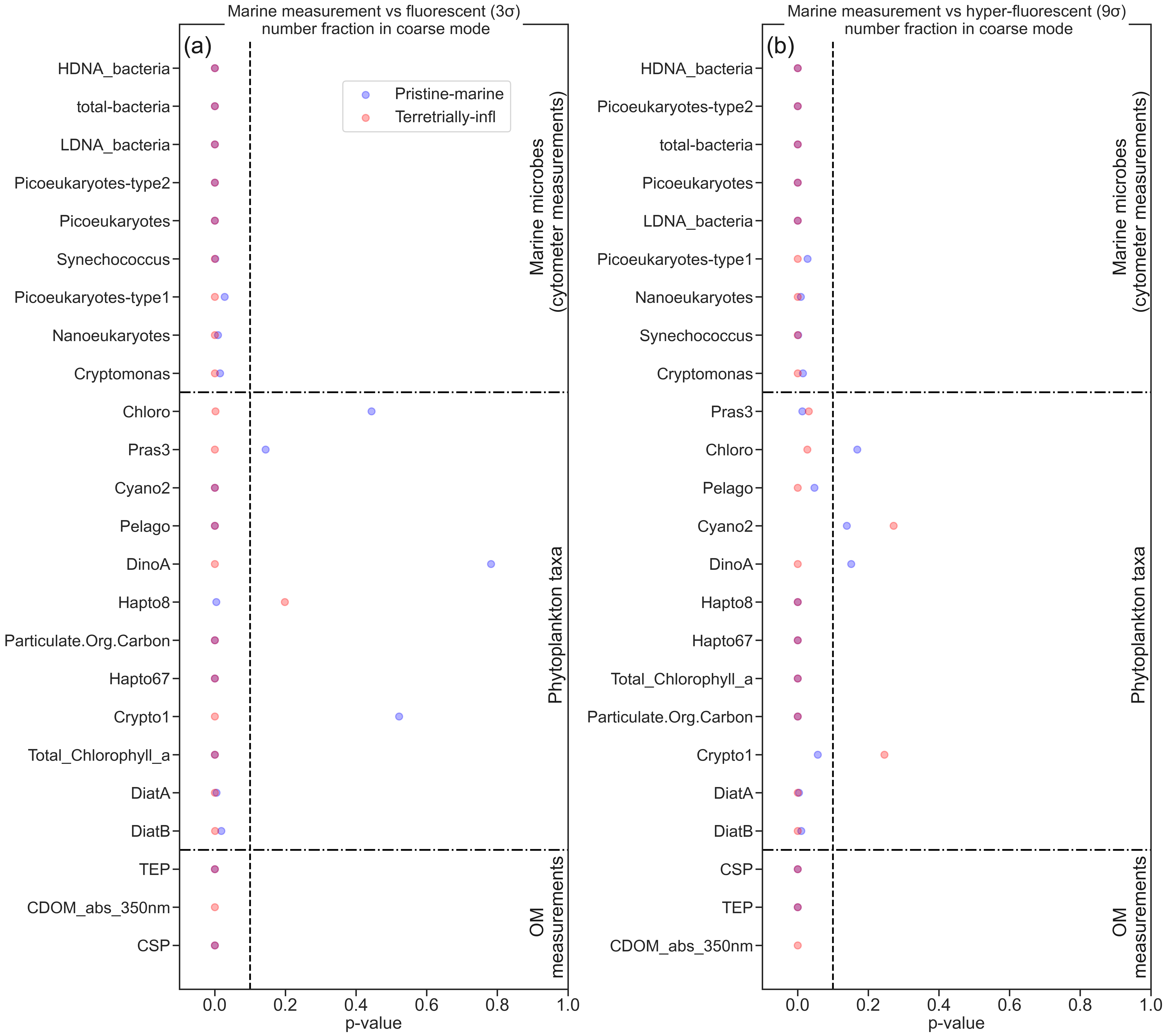


Figure S18. (a) p values of marine variables against fluorescent aerosols, and (b) p values of marine variables against hyper-fluorescent aerosols.

# Text S8: Subsampling analysis of fluorescent type classification

## S.8.1 Variation of the fluorescent type fraction of pristine-marine segment samples based on 24 hour random data points subsampling

Figure S.19 to S.21 demonstrate the resampling results for segment 1 to 3. Random subsamples of 288 points (equivalent to 24 hours of data) from 5 min time average datasets of fluorescent aerosol measurements from pristine-marine air masses of different segments were drawn. The resampling process was repeated 15 times to provide a number of resample ensembles to compare their variability with results of full segment data sets.


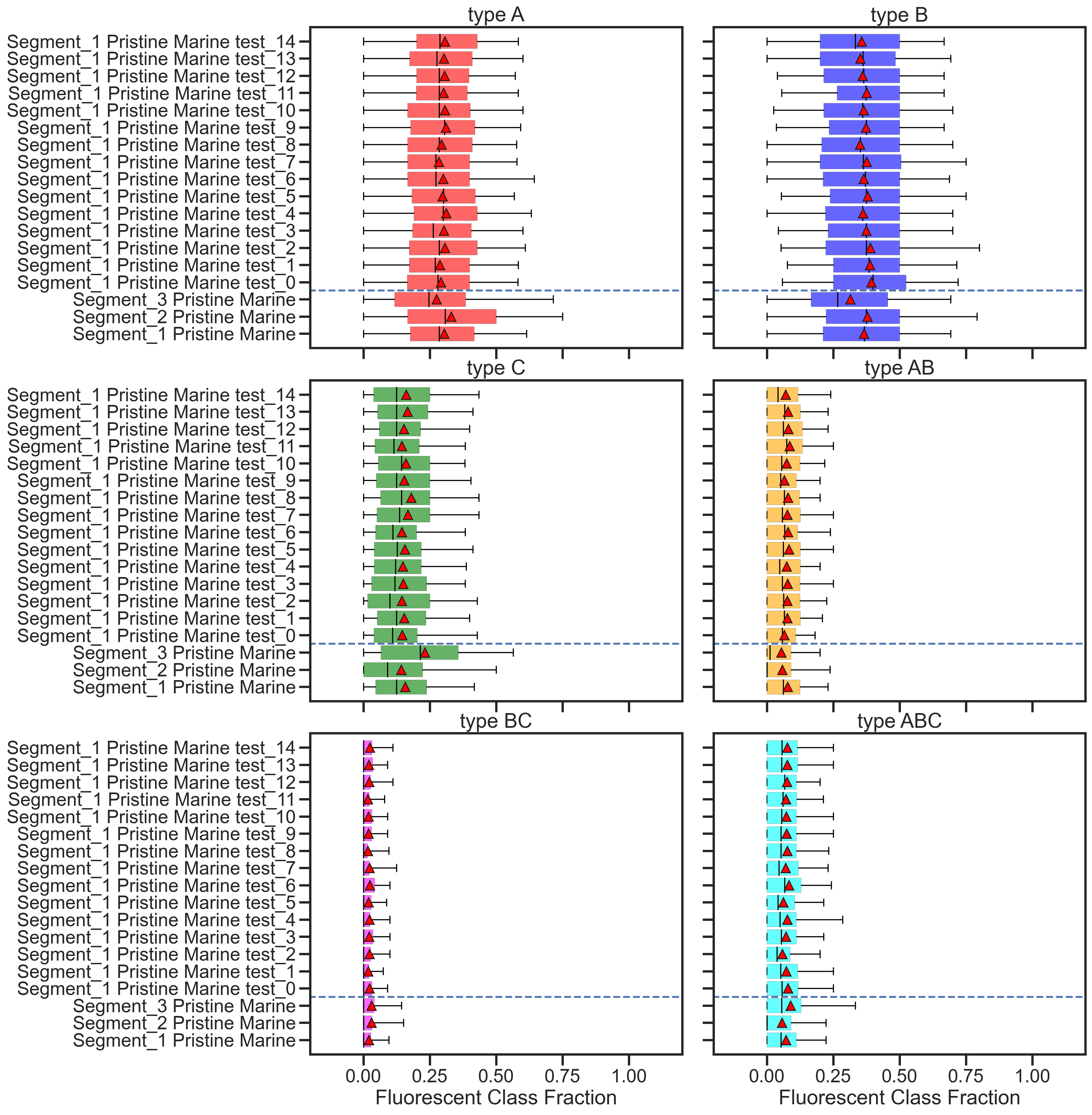


Figure S19. Fluorescent type fraction subsampling results for pristine-marine air masses from segment 1 for coarse fluorescent particles (3σ)


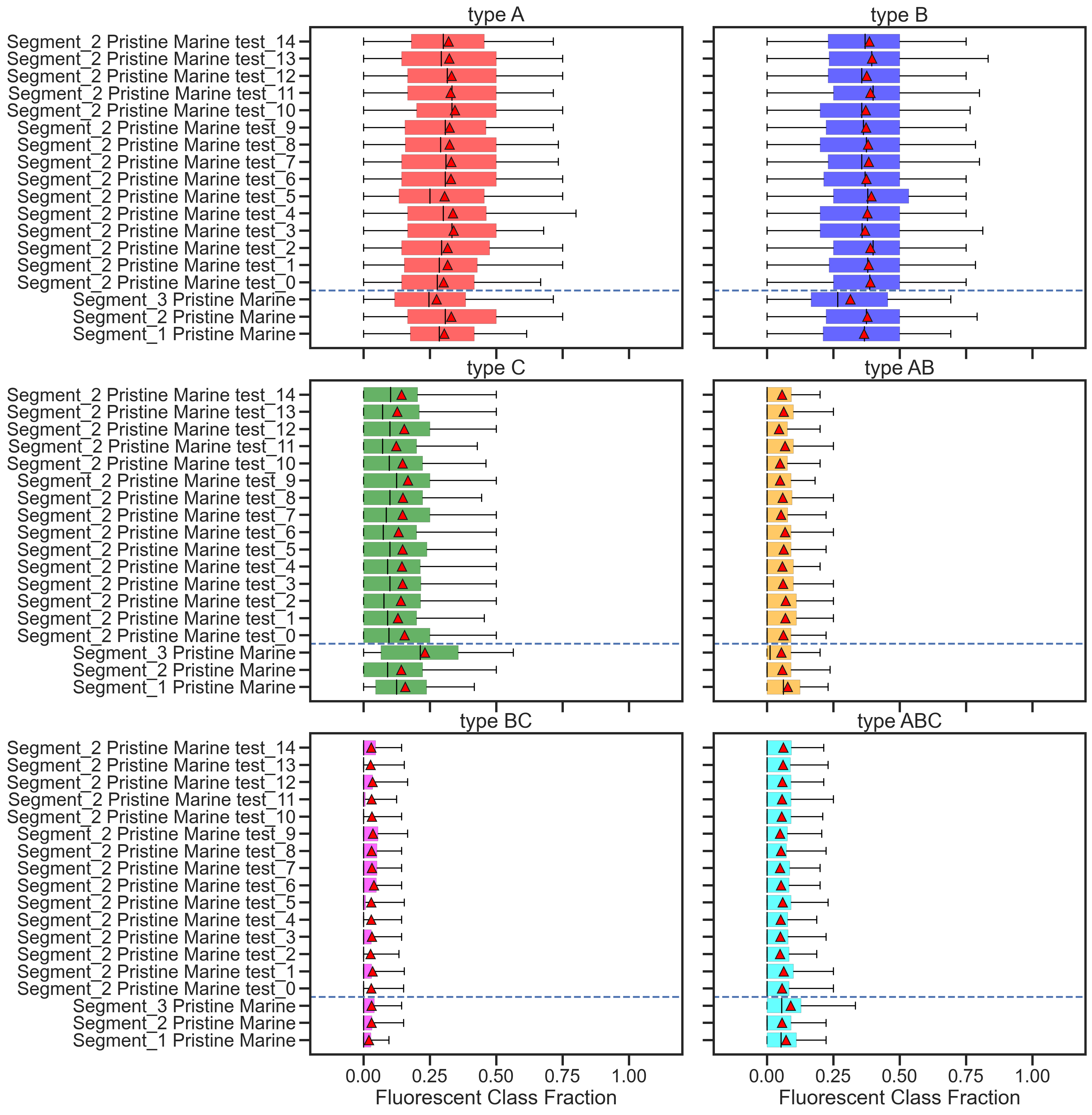


Figure S20. Fluorescent type fraction subsampling results for pristine-marine air masses from segment 2 for coarse fluorescent particles (3σ)


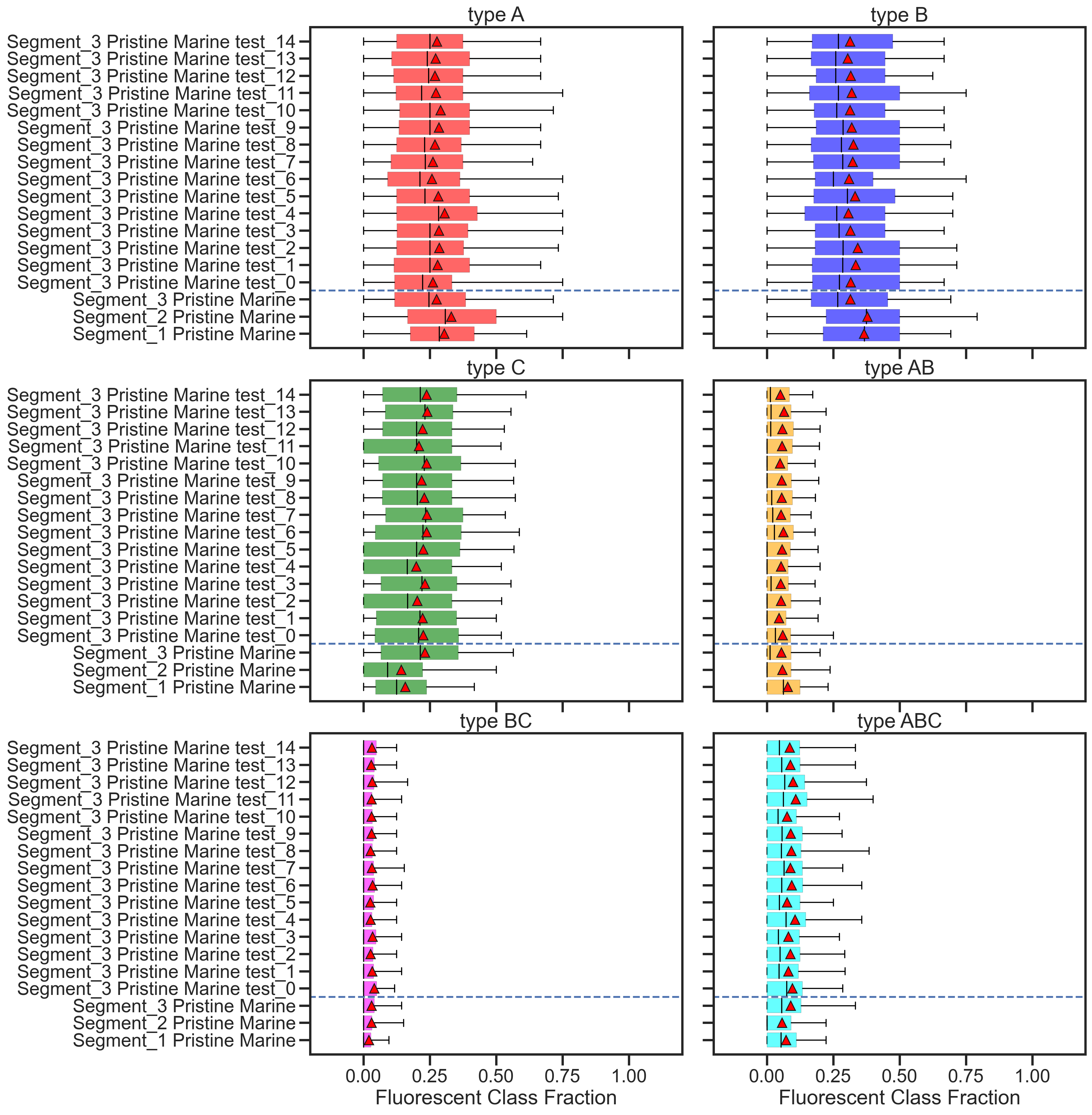


Figure S21. Fluorescent type fraction subsampling results for pristine-marine air masses from segment 3

## S.8.2 Variation of fluorescent type fraction based on a constant time window of 24 hours

To investigate the variability of fluorescent type fraction of pristine-marine air masses of each segment over different time periods, an additional subsampling analysis was conducted by drawing subsamples from a fixed time interval of 24 hours. Figure S.22 to S.24 demonstrate the resampling results for segment 1 to 3. For this analysis, in each segment 15 different and randomly selected time intervals were used. Only time intervals containing a total number of data points equivalent to or longer than 12 hours within the 24 hours were considered.


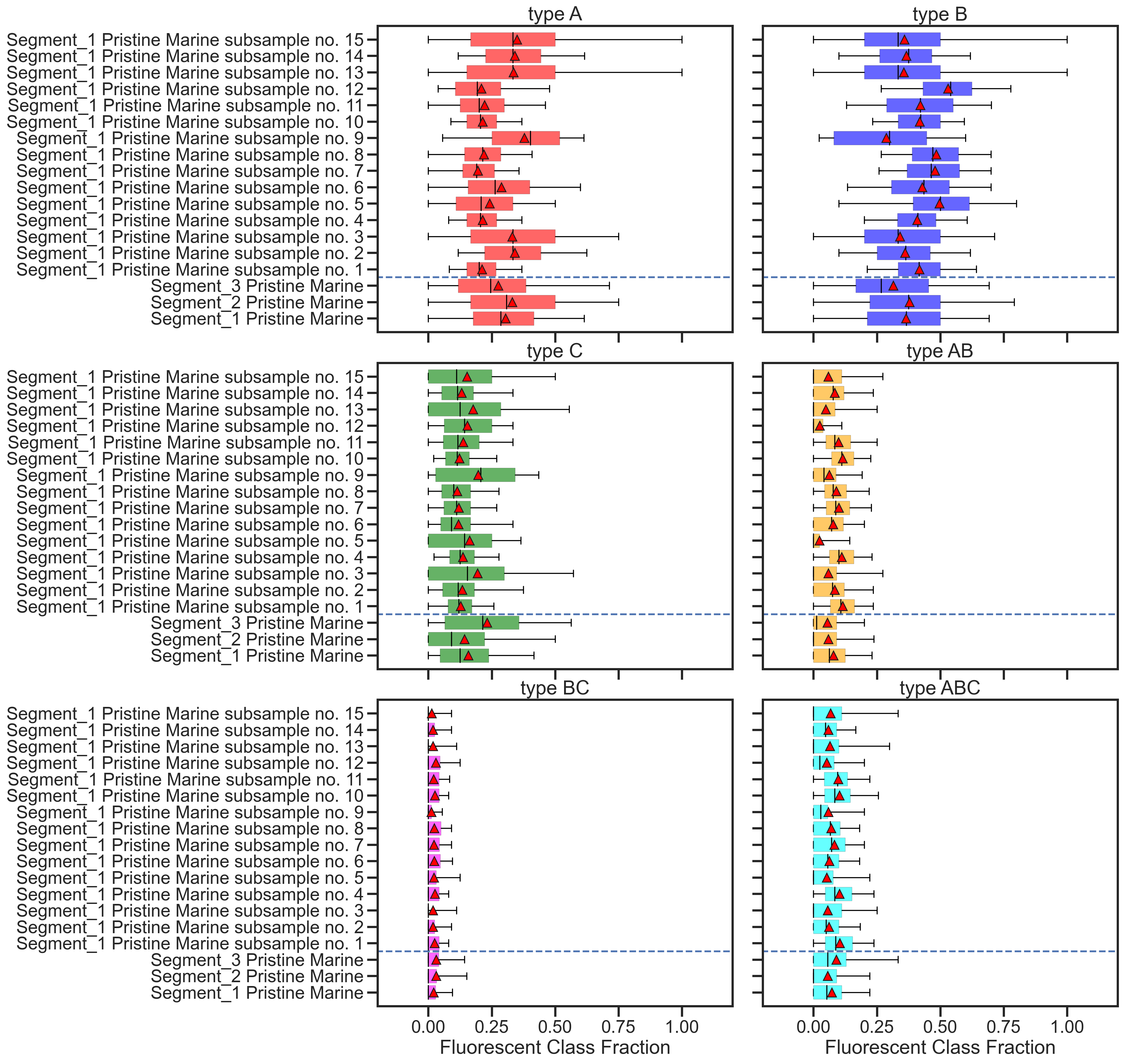


Figure S22. Results of fluorescent type fraction subsampling (based on fixed time windows) results for pristine-marine air masses from segment 1 for coarse fluorescent particles (3σ)


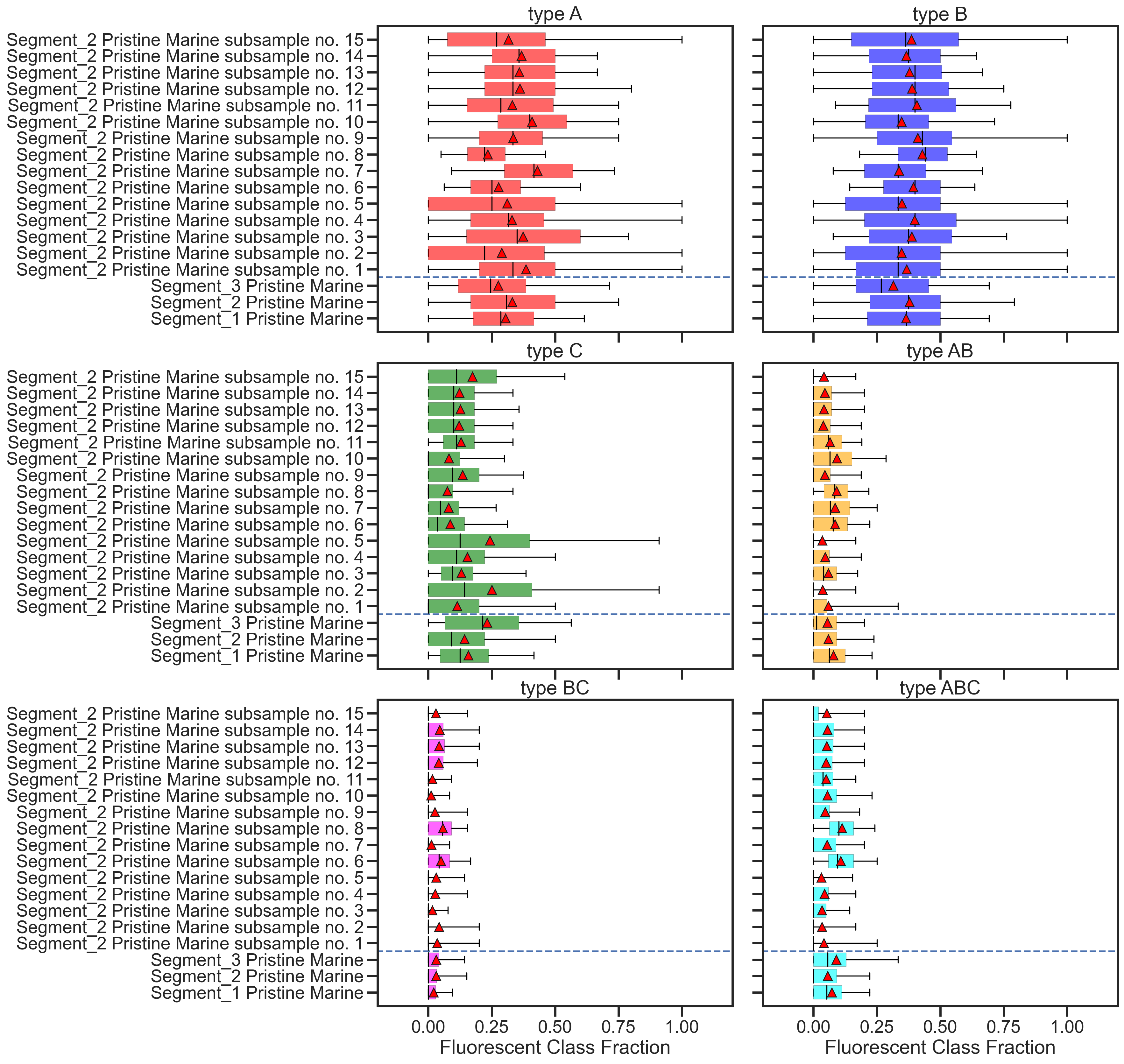


Figure S23. Results of fluorescent type fraction subsampling (based on fixed time windows) results for pristine-marine air masses from segment 2 for coarse fluorescent particles (3σ)


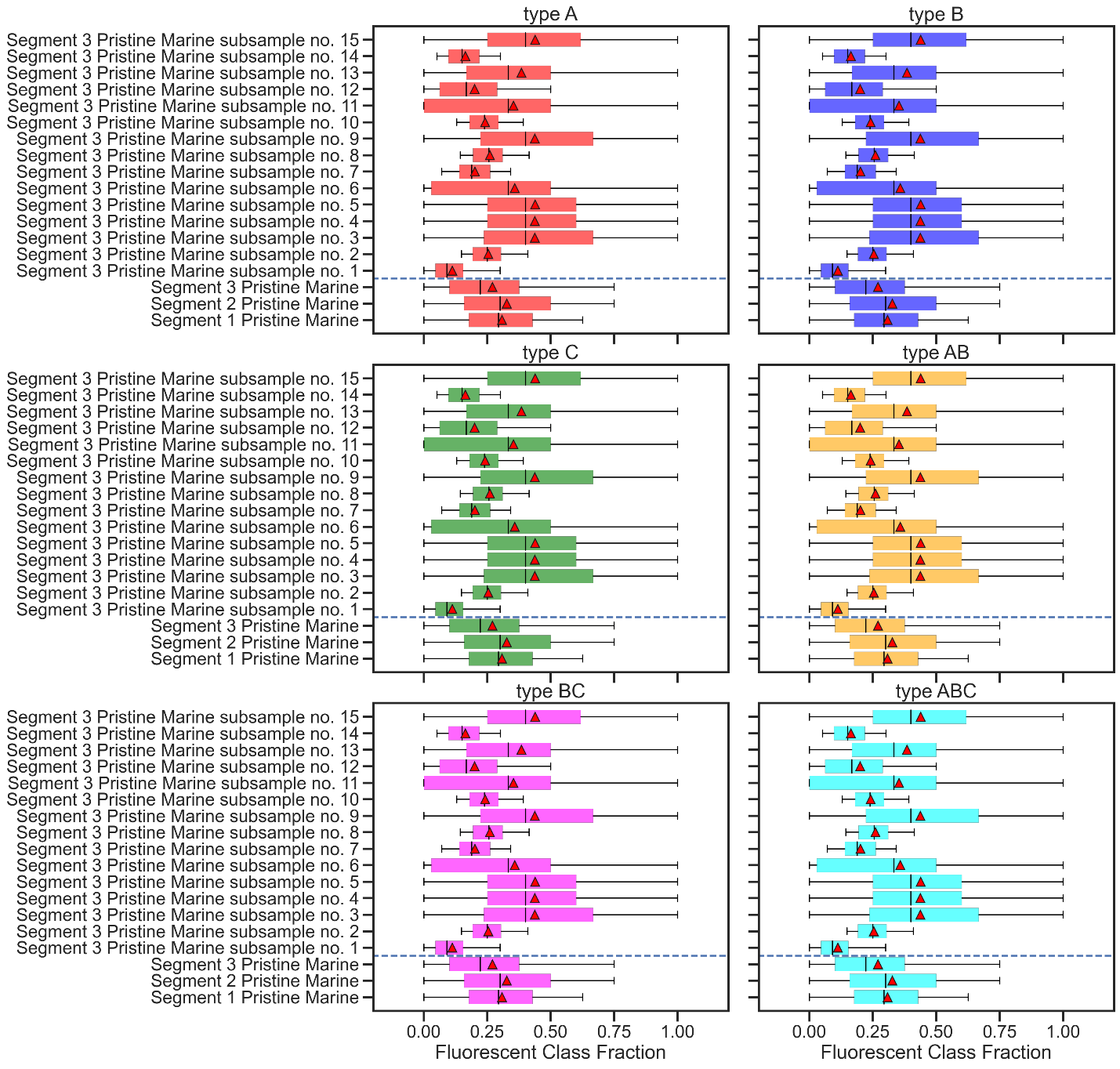
 Figure S24. Results of fluorescent type fraction subsampling (based on fixed time windows) for pristine-marine air masses from segment 3 for coarse fluorescent particles (3σ)

# Text S9: Average size distribution of aerosol fluorescent classes


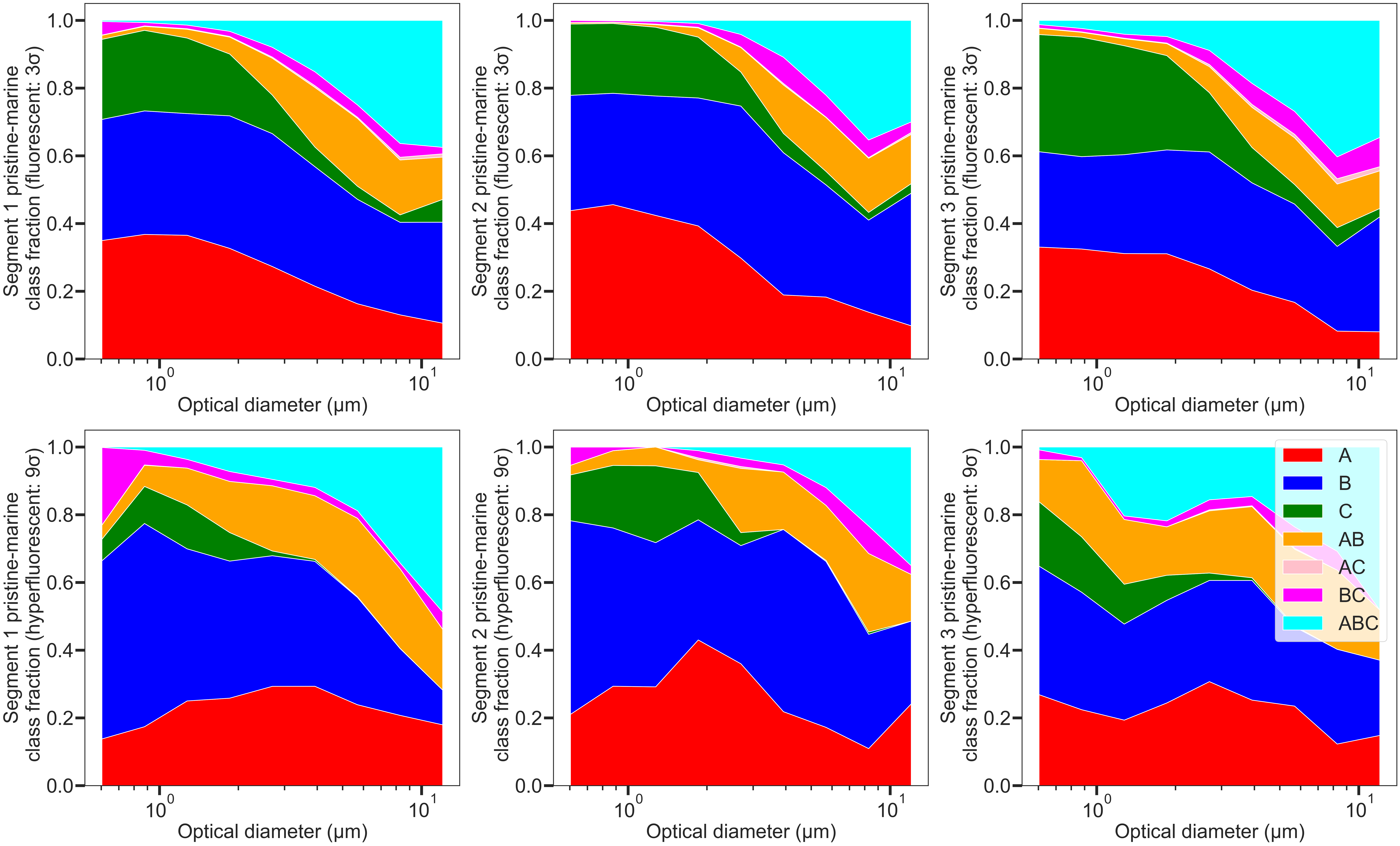


Figure S25. Size distribution of fluorescent type fraction for fluorescent particles (3σ) (top row) and hyper-fluorescent particles (9σ) bottom row for pristine-marine air masses from segment 1 to 3
