# Supplementary material for: Sources, Occurrence and Characteristics of Fluorescent Biological Aerosol Particles Measured Over the Pristine Southern Ocean
Source: J Geophys Res Atmos. 2021 Jun 9;126(11):e2021JD034811. doi: 10.1029/2021JD034811 (PMC8244095; doi:10.1029/2021JD034811)
Supplement: Supplementary file 2 [file JGRD-126-e2021JD034811-s001.zip › Data archive/_Asymmetry_Factor_Data/README.odt]

Dataset title: 

Probability density function (PDF) values of asymmetry factors (AF) at different size ranges from data collected during ACE-SPACE

Dataset abstract:

The data files AF_hist_Segment_1.csv, AF_hist_Segment_2.csv, and AF_hist_Segment_3.csv, contain the AF probability density values of pristine marine samples from segments 1, 2, and 3 of the campaign, respectively. These values are 

*****data set description*****

The PDF data is obtained by analysing the AF values of the single particle data collected by WIBS-4A during ACE-SPACE. The analysed data corresponds only to the pristine marine samples. Each of the csv files are comma separated and consist of the following headers: 

- 'AF_hist_bin': This column contains the values of AF in the PDF histogram, this represent the x-axis values of Figure 8 of Moallemi et al. (2021) manuscript.  

- 'Total Particles 0.8-1 um': This column contains the AF PDF values of total aerosol particles with optical dimeters between 0.8 to 1 μm measured by WIBS 

- 'Total Particles 1-2.5 um': This column contains the AF PDF values of total aerosol particles with optical dimeters between 1 to 2.5 μm measured by WIBS

- 'Total Particles 2.5-5 um': This column contains the AF PDF values of total aerosol particles with optical dimeters between 2.5 to 5 μm measured by WIBS

- 'Total Particles 5-14 um': This column contains the AF PDF values of total aerosol particles with optical dimeters between 5 to 14 μm measured by WIBS

- 'Fluorescent Particles 0.8-1 um': This column contains the AF PDF values of fluorescent aerosol particles with optical dimeters between 0.8 to 1 μm measured by WIBS

- 'Fluorescent Particles 1-2.5 um': This column contains the AF PDF values of fluorescent aerosol particles with optical dimeters between 1 to 2.5 μm measured by WIBS

- 'Fluorescent Particles 2.5-5 um': This column contains the AF PDF values of fluorescent aerosol particles with optical dimeters between 2.5 to 5 μm measured by WIBS

- 'Fluorescent Particles 5-14 um': This column contains the AF PDF values of fluorescent aerosol particles with optical dimeters between 5 to 14 μm measured by WIBS

- 'Hyper-fluorescent Particles 0.8-1 um': This column contains the AF PDF values of hyper-fluorescent aerosol particles with optical dimeters between 0.8 to 1 μm measured by WIBS

- 'Hyper-fluorescent Particles 1-2.5 um': This column contains the AF PDF values of hyper-fluorescent aerosol particles with optical dimeters between 1 to 2.5 μm measured by WIBS

- 'Hyper-fluorescent Particles 2.5-5 um': This column contains the AF PDF values of hyper-fluorescent aerosol particles with optical dimeters between 2.5 to 5 μm measured by WIBS

- 'Hyper-fluorescent Particles 5-14 um': This column contains the AF PDF values of hyper-fluorescent aerosol particles with optical dimeters between 5 to 14 μm measured by WIBS

Reference

Moallemi, Alireza, et al. "Sources, Occurrence and Characteristics of Fluorescent Biological Aerosol Particles Measured over the Pristine Southern Ocean." Journal of Geophysical Research: Atmospheres (2021): e2021JD034811.
