# Supplementary material for: Sources, Occurrence and Characteristics of Fluorescent Biological Aerosol Particles Measured Over the Pristine Southern Ocean
Source: J Geophys Res Atmos. 2021 Jun 9;126(11):e2021JD034811. doi: 10.1029/2021JD034811 (PMC8244095; doi:10.1029/2021JD034811)
Supplement: Supplementary file 2 [file JGRD-126-e2021JD034811-s001.zip › Data archive/_Fluorescent_ABC_class_fraction_5min_time_resolution/README.odt]

Dataset title:

Fraction of ABC classes of fluorescent and hyper-fluorescent aerosol particles from samples collected during the Antarctic Circumnavigation Expedition

Dataset abstract:

The data files ABC_frac_3std_5min_event_labeled_new.csv and ABC_frac_9std_5min_event_labeled_new.csv contain the time series of the fraction ABC classes of fluorescent and hyper-fluorescent aerosol particles respectively. The time series contain data collected during the ACE-SPACE campaign and samples collected during the return route from Cape Town to Europe. The datasets represent the ABC classification of aerosol particles with optical diameters larger than 1 μm. These time series are the 5 min average of the single particle data. In addition to the time stamp and ABC fraction, other auxiliary columns that are used for data filtering and sample categorizations are included. 

*****ABC fraction data description*****

The 5 min time series were obtained by resampling the single particle data collected by WIBS-4A instrument (University of Hertfordshire, Hatfield, UK) to 5 min time intervals and taking their mean values. The single particle data was stored as csv files and a processing code in Python was used to determine whether each single particle data if (hyper-)fluorescent and specify its ABC class. The processed single particle data was then resampled to obtain the 5 min data.  

Each of the ABC fraction files contain the following columns related to time stamp and ABC class fractions.

- time_ : UTC time stamp of the ABC fraction data

- N_A_super_1um: fraction of type A (hyper-)fluorescent aerosol particles larger than 1 μm

- N_B_super_1um: fraction of type B (hyper-)fluorescent aerosol particles larger than 1 μm

- N_C_super_1um: fraction of type C (hyper-)fluorescent aerosol particles larger than 1 μm

- N_AB_super_1um: fraction of type AB (hyper-)fluorescent aerosol particles larger than 1 μm

- N_AC_super_1um: fraction of type AC (hyper-)fluorescent aerosol particles larger than 1 μm

- N_BC_super_1um: fraction of type BC (hyper-)fluorescent aerosol particles larger than 1 μm

- N_ABC_super_1um: fraction of type ABC (hyper-)fluorescent aerosol particles larger than 1 μm

***** Auxiliary columns and filters*****

To help with the reproducibility of the data five columns were included contain relevant information and filters that has to be applied on the data. The description of the auxiliary columns are included in the following: 

- mask_5min: this column contains information on whether the sample is contaminated by Ship exhaust or not. In this column the values of 1 represent samples that are not contaminated by the ship exhaust and are included in our analysis in the paper. The nan values in this column represent the contaminated samples

- dist_to_land_m_new: this column contains the distance of the ship from landmasses (continents and islands) in unit of m

- shore_label: this column contains binary information. The value of 1 represent samples that are further than 200 km from landmasses (pristine marine sample category) and value of 0 represent samples that are closer than 200 km to land masses (terrestrially-influenced)

- region_label: this column contains label of the regions where samples were collected. The included regions correspond to the segment where the sample are included and whether they are pristine marine, terrestrially-influenced, or whether they are part of near land events specified in the manuscript.
