# Supplementary material for: Sources, Occurrence and Characteristics of Fluorescent Biological Aerosol Particles Measured Over the Pristine Southern Ocean
Source: J Geophys Res Atmos. 2021 Jun 9;126(11):e2021JD034811. doi: 10.1029/2021JD034811 (PMC8244095; doi:10.1029/2021JD034811)
Supplement: Supplementary file 2 [file JGRD-126-e2021JD034811-s001.zip › Data archive/_Fluorescent_number_concentration_merged_with_marine_variables/README.odt]

Dataset title: 

Fluorescent and hyper-fluorescent aerosol particles concentration and fraction and marine variables collected during the Antarctic Circumnavigation Expedition

Dataset abstract:

The data files WIBS_1H_with_marine_var.csv contains the 1 hour time series of number concertation and fraction of fluorescent and hyper-fluorescent and marine variables used in this study. The aerosol particles included here are have optical diameters larger than 1 μm.

*****data description*****

The hourly time series of fluorescent aerosols were obtained by resampling the 5 min time series of fluorescent and hyper-fluorescent data. The Marine data was then merged with the hourly fluorescent data.    

The data file contain the following columns

- time_ : UTC time stamp of the ABC fraction data

- 'Norm_flu_coarse_3std': fraction of fluorescent aerosol particles to total aerosol particles

- 'Norm_flu_coarse_9std': fraction of hyper-fluorescent aerosol particles to total aerosol particles

- 'N_super_sum_3std': number concentration of fluorescent aerosol particles (#/l)

- 'N_super_sum_9std': number concentration of hyper-fluorescent aerosol particles (#/l)

- 'HDNA_bacteria': Concentration of high DNA containing bacteria (Cells mL-1)

- 'total-bacteria': Concentration of total bacteria (Cells mL-1)

- 'LDNA_bacteria': Concentration of low DNA containing bacteria (Cells mL-1)

- 'Picoeukaryotes-type2': Concentration of picoeukaryote type 2 cells (Cells mL-1)

- 'Picoeukaryotes': Concentration of picoeukaryote (type 1 & type 2) cells (Cells mL-1)

- 'Synechococcus': Concentration of Synechoccocus sp. Cells (Cells mL-1)

- 'Picoeukaryotes-type1': Concentration of picoeukaryote type 1 cells (Cells mL-1)

- 'Nanoeukaryotes': Concentration of nanoeukaryote cells (Cells mL-1)

- 'Cryptomonas': Concentration of cryptomonas cells (Cells mL-1)

- 'Chloro': Chlorophyte contribution to chlorophyll biomass (μg L-1)

- 'Crypto1': Cryptophyte contribution to chlorophyll biomass (μg L-1)

- 'Cyano2': Cyanobacteria type 2 contribution to chlorophyll biomas (μg L-1)

- 'DiatA': Diatom type contribution to chlorophyll biomas (μg L-1)

- 'DiatB': Diatom type 2 contribution to chlorophyll biomas (μg L-1)

- 'DinoA': Diatom type 2 contribution to chlorophyll biomas (μg L-1)

- 'Hapto8': Haptophyte type 8 contribution to chlorophyll biomas (μg L-1)

- 'Hapto67': Haptophyte type 6&7 contribution to chlorophyll biomas (μg L-1)

- 'Pras3': Prasinophyte type 3 contribution to chlorophyll biomas (μg L-1)

- 'Pelago': Pelagophyte contribution to chlorophyll biomas (μg L-1)

- 'Total_Chlorophyll_a': Total chlorophyll-a concentration (μg L-1)

- 'Particulate.Org.Carbon': Particulate organic carbon concentration (μM)

- 'CDOM_abs_350nm': Colored dissolved organic material (CDOM) absorption  at 350 nm (m-1)

- 'TEP': Transparent Exopolymeric Particles (µg XG eq L-1)

- 'CSP': Coomasie Stainable Particles (µg BSA eq L-1)

- 'shore_label': this column contains binary information. The value of 1 represent samples that are further than 200 km from landmasses (pristine marine sample category) and value of 0 represent samples that are closer than 200 km to land masses (terrestrially-influenced)
