# Supplementary material for: Sources, Occurrence and Characteristics of Fluorescent Biological Aerosol Particles Measured Over the Pristine Southern Ocean
Source: J Geophys Res Atmos. 2021 Jun 9;126(11):e2021JD034811. doi: 10.1029/2021JD034811 (PMC8244095; doi:10.1029/2021JD034811)
Supplement: Supplementary file 2 [file JGRD-126-e2021JD034811-s001.zip › Data archive/_Fluorescent_number_concentration_merged_with_SSA/README.odt]

Dataset title: 

Fluorescent and hyper-fluorescent aerosol particles concentration and fraction, coordinates and SSA proxy data collected during ACE-SPACE campaign

Dataset abstract:

The data files WIBS_1H_data_merged_with_SSA_proxies.csv and WIBS_24H_data_merged_with_Na_data.csv contain the 1-hour and 24-hour time series of number concertation of fluorescent and hyper-fluorescent and different sea spray aerosol particle proxies.

*****data description for WIBS_1H_data_merged_with_SSA_proxies.csv*****

The hourly time series of fluorescent aerosols were obtained by resampling the 5 min time series of fluorescent and hyper-fluorescent data to 1 hour and take the mean values. The SSA proxies form other measurement during ACE-SPACE were merged with the 1-hour fluorescent time series data to construct this dataset. 

The data file contain the following columns

- 'time_': UTC time stamp of the ABC fraction data

- 'N_tot_g1000': number concentration of total particles measured by WIBS (#/l)

- 'N_super_sum_9std': number hyper-fluorescent particles measured by WIBS (#/l)

- 'N_super_sum_3std': number fluorescent particles measured by WIBS (#/l)

- 'Norm_flu_coarse_9std': fraction of hyper-fluorescent to total aerosol particles 

- 'Norm_flu_coarse_3std': fraction of fluorescent to total aerosol particles

- 'latitude': the latitude of sampled point

- 'longitude': the longitude of sampled point

- 'Cl_ACSM': concentration of chloride (μg m-3)

- 'u10': air wind speed (m/s)

- 'shore_label': this column contains binary information. The value of 1 represent samples that are further than 200 km from landmasses (pristine marine sample category) and value of 0 represent samples that are closer than 200 km to land masses (terrestrially-influenced)

- 'region_label': this column contains label of the regions where samples were collected. The included regions correspond to the segment where the sample are included and whether they are pristine marine, terrestrially-influenced, or whether they are part of near land events specified in the manuscript.

*****data description for WIBS_24H_data_merged_with_Na_data.csv *****

The 24-hour time series of fluorescent aerosols were obtained by resampling the 1 hour dataset time series of fluorescent and hyper-fluorescent data to 24 hour and take the mean values. This was done since the Na+ measurement were conducted in 24-hour time intervals. 

- 'time_': UTC time stamp of the ABC fraction data

- 'N_tot_g1000': number concentration of total particles measured by WIBS (#/l)

- 'N_super_sum_9std': number hyper-fluorescent particles measured by WIBS (#/l)

- 'N_super_sum_3std': number fluorescent particles measured by WIBS (#/l)

- 'shore_label': this column contains binary information. The value of 1 represent samples that are further than 200 km from landmasses (pristine marine sample category) and value of 0 represent samples that are closer than 200 km to land masses (terrestrially-influenced)

- 'Na+': mass concentration of Na+ (μg m-3)
