# Supplementary material for: Sources, Occurrence and Characteristics of Fluorescent Biological Aerosol Particles Measured Over the Pristine Southern Ocean
Source: J Geophys Res Atmos. 2021 Jun 9;126(11):e2021JD034811. doi: 10.1029/2021JD034811 (PMC8244095; doi:10.1029/2021JD034811)
Supplement: Supplementary file 2 [file JGRD-126-e2021JD034811-s001.zip › Data archive/_List_of_events/README.odt]

Dataset title: 

List of time intervals of near land high fluorescent aerosol events during ACE-SPACE and in West African region 

Dataset abstract:

This csv file contains the starting and end times in UTC of the events specified in the manuscript

*****data description for WIBS_1H_data_merged_with_SSA_proxies.csv*****

The csv file contains the starting and end time of the following events:

'(Pristine SO Island) Kerguelen'

'Hobart',

'(Pristine Antarctic Region) Mertz',

'(Pristine Antarctic Region) Young',

'(Pristine Antarctic Region) Siple', 'Punta Arenas',

'(Pristine SO Island) South Georgia', '(Pristine SO Island) Bouvet',

'West African Dust event'

The starting and end points are in UTC
