# Supplementary material for: Sources, Occurrence and Characteristics of Fluorescent Biological Aerosol Particles Measured Over the Pristine Southern Ocean
Source: J Geophys Res Atmos. 2021 Jun 9;126(11):e2021JD034811. doi: 10.1029/2021JD034811 (PMC8244095; doi:10.1029/2021JD034811)
Supplement: Supplementary file 2 [file JGRD-126-e2021JD034811-s001.zip › Data archive/_Particle_size_distribution_data/README.odt]

Dataset title: 

Particle number size distribution of (hyper-)fluorescent and total aerosol particles measured during ACE-SPACE 

Dataset abstract:

The data files contain the time series of number size distribution of fluorescent, hyper-fluorescent particles, and their ABC classes and total aerosol particles.

*****data description for WIBS_1H_data_merged_with_SSA_proxies.csv*****

The size distributions time series have mainly time resolutions of 5 min. The size distribution was obtained by groping the single particle data into 5 min time windows and obtain the size distribution statistics for (hyper-)fluorescent, ABC classes and total aerosol particles, using a python programs. The files containing 3std in their names corresponds to fluorescent aerosol data while files with 9std in their name corresponds to hyper-fluorescent aerosol particles. The files with A, B, C, AB, AC, BC, ABC in their names indicate that the size distribution are related to specific ABC classes. PSD_all_flu_3std_clean_5min_9bin_0.5um_14um.csv, PSD_all_flu_9std_clean_5min_9bin_0.5um_14um.csv, and 

PSD_tot_clean_5min_9bin_0.5um_14um.csv, correspond to sum of all fluorescent, sum of all hyper-fluorescent and total aerosol particles, respectively. In our analysis we had 9 size bins which are part of the header of the CSV files. The size bins are the followings:

0.6028570409756517 (μm), 0.8764012816178796 (μm), 1.2740652496625366 (μm), 1.8521678304726774 (μm), 2.6925824035672523 (μm), 3.914332103559852 (μm), 5.69044638955526 (μm), 8.272466223025312 (μm), 12.02606838308921 (μm)

Note that PSD_all_flu_3std_clean_9bin_0.5um_14um_12hour_ave, PSD_all_flu_9std_clean_9bin_0.5um_14um_12hour_ave, PSD_tot_clean_9bin_0.5um_14um_12hour_ave.csv, correspond to sum of all fluorescent, sum of all hyper-fluorescent and total aerosol particles, respectively, that obtained by resampling the 5 min data to 12 hour time resolutions and taking the mean. The 12 hour time resolutions data is used in Figure 7 of the Moallemi et al. (2021) manuscript. 

The columns in data files:  

- 'time_': UTC time stamp of sample

- 0.6028570409756517 : size distribution data in 0.6028570409756517 μm size bin

- 0.8764012816178796 : size distribution data in 0.8764012816178796 μm size bin

- 1.2740652496625366 : size distribution data in 1.2740652496625366 μm size bin

- 1.8521678304726774 : size distribution data in 1.8521678304726774 μm size bin

- 2.6925824035672523 : size distribution data in 2.6925824035672523 μm size bin

- 3.914332103559852 : size distribution data in 3.914332103559852 μm size bin

- 5.69044638955526 : size distribution data in 5.69044638955526 μm size bin

- 8.272466223025312 : size distribution data in 8.272466223025312 μm size bin

- 12.02606838308921 : size distribution data in 12.02606838308921 μm size bin

- 'region_label': this column contains label of the regions where samples were collected. The included regions correspond to the segment where the sample are included and whether they are pristine marine, terrestrially-influenced, or whether they are part of near land events specified in the manuscript.

Reference

Moallemi, Alireza, et al. "Sources, Occurrence and Characteristics of Fluorescent Biological Aerosol Particles Measured over the Pristine Southern Ocean." Journal of Geophysical Research: Atmospheres (2021): e2021JD034811.
