# Supplementary material for: Sources, Occurrence and Characteristics of Fluorescent Biological Aerosol Particles Measured Over the Pristine Southern Ocean
Source: J Geophys Res Atmos. 2021 Jun 9;126(11):e2021JD034811. doi: 10.1029/2021JD034811 (PMC8244095; doi:10.1029/2021JD034811)
Supplement: Supplementary file 2 [file JGRD-126-e2021JD034811-s001.zip › Data archive/_RA2B_data/README.odt]

Dataset title: 

Single particle RB2A values from fluorescent aerosol measurements during ACE-SPACE campaign and West African event

Dataset abstract:

The data files R_B2A_3std.csv and R_B2A_9std.csv contain the single particle time series of RB2A for fluorescent and hyper-fluorescent particles. The data included time periods of pristine marine samples and near land fluorescent aerosol events.

*****data description *****

The RB2A data was obtained from analysis of single particle measurements conducted by WIBS-4A during the camping. In Moallemi et al. (2021) the detailed description of the analysis is mentioned. 

The csv files contains the following columns:

- 'time_': UTC time stamp of the ABC fraction data

- 'region_label': this column contains label of the regions where samples were collected. The included regions correspond to the segment where the sample are included and whether they are pristine marine, terrestrially-influenced, or whether they are part of near land events specified in the manuscript.

- 'R_B2A': The values of RB2A

Reference

Moallemi, Alireza, et al. "Sources, Occurrence and Characteristics of Fluorescent Biological Aerosol Particles Measured over the Pristine Southern Ocean." Journal of Geophysical Research: Atmospheres (2021): e2021JD034811.
